# *Salmonella* Typhimurium Triggered Unilateral Epididymo-Orchitis and Splenomegaly in a Holstein Bull in Assiut, Egypt: A Case Report

**DOI:** 10.3390/pathogens9040314

**Published:** 2020-04-24

**Authors:** Manal A. M. Mahmoud, Gaber Megahed, Mohamed S. Yousef, Fatma Abo Zakaib Ali, Rania S. Zaki, Hanan H. Abdelhafeez

**Affiliations:** 1 Department of Animal Hygiene, Faculty of Veterinary Medicine, Assiut University, 71526 Assiut, Egypt; manalmahmoud@vet.au.edu.eg; 2 Department of Theriogenology, Faculty of Veterinary Medicine, Assiut University, 71526 Assiut, Egypt; gabermegahed@vet.au.edu.eg (G.M.); Samy_Yousef@vet.aun.edu.eg (M.S.Y.); 3 Department of Pathology and Clinical Pathology, Faculty of Veterinary Medicine, Sohag, University, 82524 Sohag, Egypt; fatma_ali@vet.sohag.edu.eg; 4 Department of Food Hygiene, Faculty of Veterinary Medicine, New Valley University, 72511 Al-Kharga, Egypt; raniasamir5555@nu.edu.eg; 5 Department of Anatomy, Embryology and Histology, Faculty of Veterinary Medicine, Assiut University, 71526 Assiut, Egypt

**Keywords:** *Salmonella* Typhimurium sequencing, *Salmonella* virulence genes (*stn*, *avr*A and *sop*B), VITEK identification, necrotizing intratubular pyogranulomatus orchitis and epididymitis

## Abstract

This report illustrates, for the first time, a case of unilateral orchitis and epididymitis in a Holstein-Friesian bull, associated with *Salmonella enterica* infection (*Salmonella enterica* serovar Typhimurium). A one and a half-year-old Holstein-Friesian bull had arrived at the Veterinary Hospital of Assiut University suffering from anorexia accompanied with persistent fever, which did not respond to oxytetracycline and flunixin meglumine injection for 15 days. Gross examination revealed left scrotal enlargement (three times its normal size), heat sensation, and induration of the testis and epididymis, which was painful on external palpation. Microbiological and pathological examinations of the left testicle, epididymis, and spleen samples were performed. *S.* Typhimurium was recovered from the affected tissues and its critical virulence genes (*stn*, *avr*A and *sop*B) were identified. Pathological examination revealed a unilateral necrotizing intratubular pyogranulomatus orchitis and epididymitis with severe peri-orchitis. In addition, splenomegaly with a firm and large whitish nodular capsular structure associated with different stages of granulomatous reaction around the white and red pulp. To the authors’ knowledge, this report is the first isolation of *S.* Typhimurium from the epididymis and testicles of a Holstein-Friesian bull. These results highlight the importance of including *S.* Typhimurium among the health disorders associated with stressful situations in bovine with orchitis and or/epididymitis. In Egypt, *Salmonella spp.* infection as being enzootic with high probability of dissemination should be considered one of genital health problems among cattle farms.

## 1. Introduction

Salmonellosis is a serious foodborne poisoning and a major public health concern, particularly in the developing countries. *Salmonella* spp. is a gram-negative, facultative anaerobic rods, which classically manifests as systemic septicemia and gastroenteritis in cattle. Additional but less common clinical presentations may include respiratory manifestations, arthritis, and abortion. *Salmonella*, after surpassing gastric defenses, can proliferate within macrophages, escape phagocytosis by neutrophils, and disperse throughout the body, causing a wide variety of clinical manifestations [1]. Latent carrier could carry the organism in the mesenteric lymph node or tonsils, and usually the reactive form of infection occurs after stress or immunosuppressant [1], and the organism largely shedding through the fecal route [2]. In warm-blooded vertebrates, salmonellosis has been linked with serovar of *S. enterica*. More than 200 antigenically distinct serotypes of *Salmonella* possess pathogenic potential [3]. *Salmonella* has been associated with livestock farm animals (primarily cattle, sheep and pigs), and their products which are collectively considered as critical sources of infection to humans [4]. The annual estimate of the morbidity and mortality of non-typhoid *Salmonella* (NTS) showed 93.8 million illnesses and 155 thousand deaths, at which African countries have the highest infection rate, respectively [5]. 

*Salmonella* Typhimurium is the second commonest *salmonella* serotype in cattle linked with abortion in the UK that is commonly associated with carrier animals or consumption of contaminated feed or water [6]. Subclinical *Salmonella* are common and may easily disseminate without recognition between animals that may become persistent carriers [7].

*Salmonella* virulence factors play a crucial role in the pathogenicity and severity of infection to the target host. The virulence genes encoding these virulence factors are partially located in a chromosomal segment locus called *Salmonella* pathogenicity island (SPI), which helps in maintaining the integrity of the membrane composition of *Salmonella* and overcoming host defenses [8]. *Salmonella avr*A is an effector protein that limits the host’s inflammatory responses through inhibition of α IL-8 and TNF- and induction of cell apoptosis, especially that of macrophages [9]. The secreted protein *sop*B is involved in epithelial cell adhesion, cytoskeletal rearrangements, and in phagocytic and non- phagocytic cell invasion, which is essential for *Salmonella* enteropathogenicity, causing acute inflammatory cell influx, intestinal fluid secretion, and enteritis that correlate with clinical diarrhea [10,11]. The biological activities of *stn* are also important for *Salmonella* enterotoxicity [12].

Orchitis or epididymitis associated with *Salmonella* infection has rarely been reported in animals. A case of unilateral suppurtaive epididymo-orchitis associated with *Salmonella enterica* subspecies *diarizonae* was described by Ferreras in a two-year-old ram [13]. However, *Salmonella spp.* were commonly isolated from suppurative testicular lesions as an extraintestinal complication of enteric salmonellosis in humans [14,15], or in unique testis association cases [16]. Genito-urinary tract involvement is infrequent and is often combined with congenital abnormalities, immunosuppression, and malignancy as reported in several cases by the New York *Salmonella* Center [2]. Infectious orchitis and epididymitis in bulls were mostly linked with bacterial (*Brucella abortus, Actinobacterium pyogenes, Mycobacterium tuberculosis*) or viral agents (Bovine Herpes Virus 1) [17] and usually accompanied by decreased fertility or infertility as well as thermal injury to the testis [18]. The objective of this report was to identify the causative agent and the histopathological health implication in a Holstein bull admitted to the veterinary hospital suffering from unilateral epididymo-orchitis in Assiut, Egypt. 

## 2. Results

### 2.1. Microbiological and Sequencing Results:

**Figure 1:**
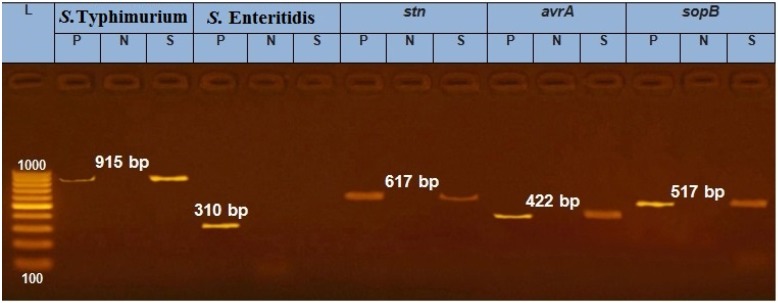
PCR image identifying *Salmonella serotype* (*S.* Typhimurium) and virulence genes (*stn*, *avr*A and *sop*B) of the testis sample from a Holstein bull. Note: P: positive reference strain, N: negative control, S: sample, DNA marker: 100–1000 bp; image representing positive amplification of 915, 617, 422 and 517 bp fragments of *S.* Typhimurium, *stn, avr*A and *sop*B, respectively.

*Salmonella enterica* serovar Typhimurium was isolated from the testicle sample of the affected bull (Figure 1). The results showed presence of different virulence genes of *Salmonella* including *stn, avr*A and *sop*B (Figure 1). The complex processes of *Salmonella spp.* virulence (adhesion, invasion and enterotoxin production) could have a major role in the pathogenicity of *Salmonella* to overcome the host defenses.

The partial sequence for 16S ribosomal RNA gene offered a better understanding and a more accurate diagnosis of the case. Our results confirmed, with 99% similarity to the GenBank database, the presence of *S. enterica subsp. enterica* serovar Typhimurium, which was the primary cause of the clinical signs observed on the infected bull. The gene bank accession number for our nucleotide sequence has been deposited as (SUB4878538 Seq MK253289).

### 2.2. Histopathological Results

#### 2.2.1. Gross Findings

**Figure 2:**
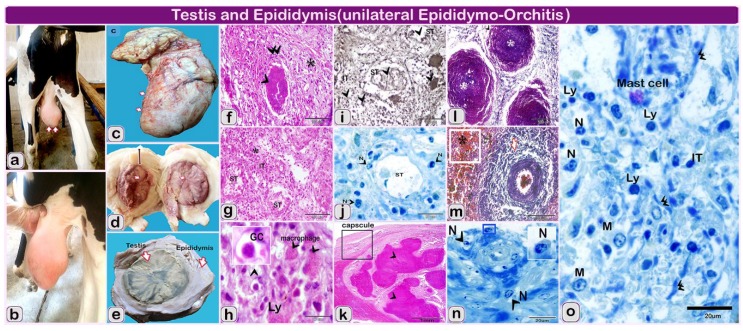
(**a**–**e**): the gross finding and hitolopathological structure of the examined testis and epididymis of Holstein-Friesian bull (one and a half-year). Note: (**a**–**e**): the gross finding of the testis, (**f**–**i**): paraffin sections of the left testis of infected bull (**f**–**h**) stained by Hematoxylin and eosin. (**i**) Sections stained by Gomori calcium method for alkaline phosphatase activity. (**j**,**o**): semi thin section stained by toluidine blue. (**k**–**m**): paraffin sections of epididymis of infected bull, (**k**) stained by Hematoxylin and eosin-l and m stained by Crossman’s trichrome stain and, N: semi thin section stained by toluidine blue of Epididymis of infected bull. (**a**,**b**) Bull showing unilateral testicular enlargement (arrows); (**c**): Morphology of left testis (arrows) showing the enlargement after slaughtering of the animal; (**d**) Markedly thickened fibrotic’ tunics (line with double arrowheads) and scattered yellow patches of necrosis in the testicle (white arrows); (**e**) Cut section in the enlarged testis and epididymis after fixation showing the greatly thickened tunics and scattered yellow patches (arrows) in the testis and epididymis; (**f**) Granulomatous inflammatory lesions (arrowhead), bordered by fibrosis (double arrowheads) and inflammatory cells (star); (**g**): The seminiferous epithelium (ST) become necrotic (**n**) and desquamates with tubular destruction and the dense inflammatory infiltrates, predominantly in the interstitial (IT), (stars); (**h**) Higher magnification showing the seminiferous tubules is obliterated and replaced by numerous inflammatory cells, lymphocytes (Ly), giant cells (GC, arrowhead), (macrophage, arrowhead) and debris; (**i**) Alkaline phosphatase reaction showing the reaction of inflammatory cells within the seminiferous tubules (ST, arrowhead) and in the interstitial (interstitial, arrowhead); (**j**) Atrophy and necrosis of seminiferous tubules (ST, arrowhead), the tubular outline is retained in the affected area, but the seminiferous epithelium is destructed and the interstitial (IT) is highly infiltrated inflammatory cells. Note: destructed neutrophil (N, arrowheads); (**k**,**l**): paraffin sections of epididymis of infected bull showing: large multifocal to coalescing pyogranulomatous structures, losing normal tubular architecture (arrowheads); (**l**): coalesced: pyogranulomatous (arrowhead, *); (**m**) Extravasated hemorrhage in the interstitial tissue (*), the blood vessels are engorged with blood (arrows); (**n**) The blood vessels with Inflammatory cellular infiltration mainly neutrophil (N, arrowheads). (**o**) The inflammatory infiltrate is comprised of lymphocytes (Ly, arrowheads), neutrophils (N), (mast cells), and macrophage cells (macrophage, arrowhead, M). Note the deposition of fibers in e (double arrowheads).

Left testis and epididymis: Gross examination of the left testicle and epididymis showed necrotic intratubular pyogranulomatus orchitis and epididymitis. Left testicle and epididymis showed unilateral scrotal swelling Figure 2a,b and thickening of the tunica vaginalis Figure 2b. The cavity of the tunica vaginalis was expanded with fibrino-purulent exudates. The necrotic parenchyma became softened by liquefaction and the organ came to be like a pus-filled cavity enclosed by a dense layer of a connective-tissue capsule Figure 2c.

On the cut section, there were dispersed yellow foci of necrosis that coalesced and gave off total testicular necrosis Figure 2d. The necrotic foci were dry, yellow in color and frequently laminated and displayed the characteristics of necrotizing and granulomatous epididymo-orchitis with severe peri-orchitis. The affected testicle turns out to be a necrotic mass wrapped within the markedly thickened tunica albuginea and vaginalis Figure 2d,e. 

**Figure 3:**
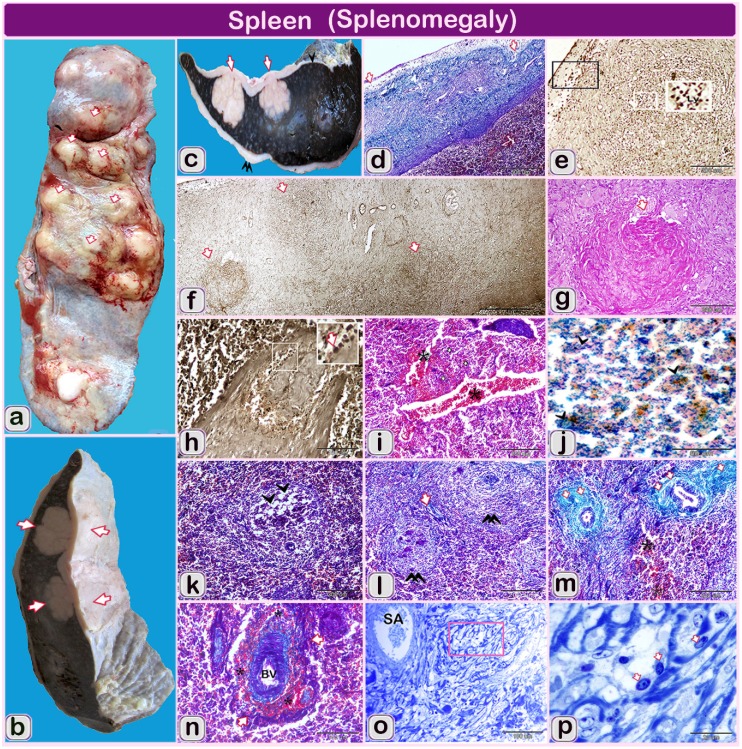
(**a**–**p**): The gross finding and hitolopathological structure of the examined spleen of Holstein-Friesian bull (one and a half-year). Note: (**a**, **b** and **c**): gross finding of the spleen (**d**,**i**,**k**,**l**,**m**,**n**) paraffin sections of the spleen of infected bull stained by Crossman’s trichrome stain. (**f**,**h**) Paraffin sections of the spleen of infected bull stained by Gomori calcium method for alkaline phosphatase activity. (**g**) Paraffin sections of the spleen of infected bull stained by Hematoxylin and eosin. (**o** and **p**) semithin sections stained by toluidine blue. (**a** and **b**): Spleen of infected bull showing enlarged, firm and nodular splenic surface (arrows); (**b**) The spleen was enlarged and increased in thickness than normal in the area of nodular structures (arrows); (**c**): The spleen of an infected bull showing large nodular masses (granulomatous structures) extended from the splenic surface till the middle of splenic parenchyma (arrowhead). Splenic surface covered by marked thickened fibrous connective tissue (double arrowheads) in some areas than other parts (arrowhead); (**d**) Segmented thick and moderately normally thickened splenic capsule (1, 2 arrows); (**e**) The alkaline phosphatase activity in lymphocytes infiltration principally at serous membrane over the capsule (Ly, arrowheads); (**f**) Stages of granuloma formation from micro-granuloma to the well-developed one (arrows); (**g**) Some granulomatous reactions formed from central Coagulative necrosis surrounded by inflammatory cells (white arrows); (**h**) Higher magnification of splenic trabeculae was thickened due to swelled and distended smooth muscular trabeculae. Blood vessel inside the splenic trabeculae was dilated. Note: diffuse lymphocytes (arrows) stained positively with alkaline phosphatase; (**i**) marked diffuse hemorrhage, the blood vessels are severely dilated and engorged with blood (*); (**j**) Diffuse hemosiderosis spots (arrowheads) of dispersed bluish precipitations of iron pigments in the white and red pulp of the spleen; (**k**) Marked fibrosis (arrows) in interstitial and around the white pulp is observed (double arrowheads); (**l**) Depletion of lymphocytes in some areas, which was characterized by reducing densities of lymphocytes (arrowheads); (**m**) Hemorrhage (*, RBCS) with red blood cells extravasated the cellular structures. Note the fibrosis around the arterial wall (arrows); (**n**) Hemorrhage (*) and existing of erythrocytes around blood vessels (BV); (**o**) Low magnification of splenic artery and showing: vacuolization in the focal area around splenic artery (SA); (**p**) Higher magnification of selected square showing vacuolization in the focal area around splenic artery (SA). Note, epithelioid cellular infiltration (arrows).

Spleen, the spleen was enlarged and increased in thickness than normal in the area of nodular structure (Figure 3a,b). The splenic surface had a firm and large whitish nodular structure resulting from the granulomatous reaction, which makes the spleen rigid, in addition to the marked thickening of the connective capsule surrounding its surface (Figure 3c).

#### 2.2.2. Microscopically

Left testis and epididymis: Histopathological examination of the testicle revealed extensive chronic inflammatory cellular infiltration Figure 2f–h resulted from *salmonella* granuloma formation ranging from micro to large granulomas Figure 2f. Inside the testicle, the seminiferous tubular epithelium of tubules and the interstitial tissues turn out to be necrotic and desquamates Figure 2g. The tubular outline remained in the affected area, but the seminiferous epithelium was destroyed Figure 2j and interchanged by numerous macrophages and multinucleated giant cells which circumscribed neutrophils and debris Figure 2h–j.

The epididymis showed marked thick tunics with organized fibrosis and diffuse inflammatory cellular infiltration Figure 2k,l. The epididymal tissue revealed large multifocal to coalescing pyogranulomas effacing normal tubular architecture Figure 2l,m. The pyogranulomas were comprised of abundant degenerate neutrophils and epithelioid macrophages surrounding extensive areas of necrosis Figure 2n,o.

Spleen: Marked inflammation in the spleen was observed including thickening of connective tissue capsule and trabeculae inter-bracketed with moderately normal thickness. The thick segments of the capsule had numerous chronic granulomatous reactions, which render the spleen rigid and nodular Figure 3d. The connective tissue capsule was infiltrated with lymph histiocytic cells Figure 3e. Different stages of granulomatous reaction were observed beginning from coagulative necrosis to complete well-developed granuloma Figure 3f,g.

Epithelioid macrophages and neutrophils diffusely infiltrated the spleen which positively stained with alkaline phosphatase. Splenic trabeculae were significantly thickened due to swollen and distended smooth muscular trabeculae in some areas Figure 3h. Hemorrhage and hemosiderosis were detected and the blood vessels were severely dilated and engorged with blood Figure 3i. Spots of hemosiderosis were stained positively with Prussian blue stain and could be seen as diffuse and disperse bluish precipitations of iron pigments in white and red pulp of spleen Figure 3j.

Inside the spleen, excessive inflammatory cellular infiltration and fibrosis particularly around the white pulp was observed. Depletion of lymphocytes in some areas in the white pulp was detected, which was characterized by reducing densities of lymphocytes Figure 3k,l. The splenic artery was constricted with protrusion the fibrous tissue sion of the endothelial cell lining in the direction of the lumen Figure 3m,n and sever thickening of the wall due to cellular hyperplasia Figure 3o,p. 

Additional macroscopic and microscopic images and their descriptions were represented in supplementary files. 

## 3. Discussion

This report describes the clinical and pathological features of bovine Salmonellosis with unilateral epididymo-orchitis associated with marked splenomegaly in a Holstein bull. This represents the first record microbiological confirmation of *S.* Typhimurium infection in a Holstein bull with concurrent involvement of the reproductive organs. Unless a traumatic origin, epididymo-orchitis due to bacterial infections always have a possibility, but *Salmonella spp.* is the least expected bacterium in inducing this phenomenon [19,20] compared with *Brucella* infection [21,22,23]. In addition, orchitis in bulls could be associated with overt abscessation, related to *Streptococcus spp., Staphylococcus spp., Arcanobacterium pyogenes, Escherichia coli, Histophilus spp., Actinomyces bovis, Actinobacillus spp.*, and *Nocardia farcinica* [24,25,26].

*Salmonella* species infection associated with orchitis was rarely reported in domestic animals [24,25,26]. A case of unilateral suppurative epididymo-orchitis associated with *Salmonella enterica subsp. diarizonae* serovar 61: k: 1, 5(7), in Spain was recorded in a two-year-old ram [27]. Moreover, *Salmonella* Abortus-Equi has been detected from testicular lesions in stallion, abortion of mare and septicemia in newborn due to ingestion of infected foodstuff [28]. However, in humans, the extraintestinal complication of salmonellosis (suppurative testicular lesions), are more frequent. *Salmonella* epidydimo-orchitis was reported from male newborn, adolescent boys and healthy old men due to nontyphoidal *Salmonella* [15,29,30]. In addition, testicular lesions due to *Salmonella* infection complication was recorded from immunocompetent or immunocompromised people [29]. About twelve cases (1.4%) of orchitis and epididymitis out of 700 cases of human extra intestinal infections caused by *Salmonella* spp. were confirmed [30]. 

However, the exact predisposing factor and mechanism for *salmonella* dissemination to testis and epididymis in bull is unclear. Generally, stressed and debilitated animals could be predisposed to salmonellosis [31]. The hematogenous route of infection in epididymo-orchitis could be considered the most probable route of infection from the intestinal tract of carrier animals [32,33]. 

Recently, *S.* Typhimurium was associated with gastroenteritis, arthritis, orchitis, oophoritis as well as, induction of granulomatous inflammation in all internal organs [34]. The extra-intestinal pathway of *Salmonella* could induce systemic inflammatory infection as splenomegaly [30,35]. Spleen is the candidate organ for trapping and destruction of blood borne pathogens. Proliferation in the phagocytes and lymphocytes numbers, along with the spreading out of immature CD71+Ter119+ reticulocytes are suggested mechanisms for responding to bacterial infection [35]. 

Virulence factors of *S.* Typhimurium could play a vital role in the pathogenicity of *Salmonella* inside the host. Their interactions with the host are complex processes that overcome host defenses [36]. Although, *Salmonella enterica* is an important diarrheal pathogen, but also infection may involve severe systemic sequel depending on serovar and host-specific factors. Genome-wide mutagenesis has indicated that enteric and systemic virulence of *Salmonella* in cattle is influenced by *Salmonella* pathogenicity island, which, encodes type III secretion system 1(T3SS-1) and (T3SS-2). These systems inject bacterial proteins into the host cells, which disrupt cellular pathways to the benefit of the pathogen. T3SS-1 promotes invasion of intestinal M cells and enterocytes by rearrangement of the subcortical actin cytoskeleton, whereas T3SS-2 facilitates the replication of intracellular bacteria within *Salmonella* containing vacuoles. Targeted mutagenesis has confirmed the role of T3SS-2 during systemic infection. It was recently reported that serovar Typhimurium promotes phagocyte motility by a process dependent on the SPI-2 gene srfH, and this was correlated with increased systemic dissemination [37], *S.* Typhimurium is taken up randomly by the different phagocytes (macrophages, dendritic cells, and poly-morphonuclear cells) and disseminates rapidly through efferent lymph in mesenteric lymph nodes and through the blood stream in spleen and liver [38]. In such bull, *S.* Typhimurium was hypothesized to disseminate to the spleen and genital organs (testis and epidedymis) through the blood stream. *The avrA* gene recovered from the affected bull was similar to that of *S.* Typhimurium isolated from dairy cattle with clinical mastitis (100% frequency) detected by Abd El- Tawab et al. [39]. Epididymo-orchitis may be attributed to changes in the ability of this serovar to adapt to new hosts and, consequently, emergence of novel virulent strains [40]. In agreement, *S.* Typhimurium infection in broilers was associated with *stn* gene (at incidence of 52.94%) [41]. The *stn* from *S.* Typhimurium strain Q1 showed enterotoxic and cytotoxic activities responsible for its virulence. The sequence of *stn* showed some similarity to the active site of cholera toxin (CT) and heat-labile enterotoxin (LT) which suggests that *stn* could act as a key factor in acute gastroenteritis and diarrhea and contributes to *Salmonella* virulence [42].

The virulence factor *sop*B is essential for *Salmonella* enteropathogenicity, causing an acute inflammatory cell influx, intestinal fluid secretion and enteritis that correlate with clinical diarrhea [10]. Similarly, all *S.* Typhimurium samples isolated from pigs carried *sop*B virulence genes as reported by Barilli et al. [43]. 

Testicular examination revealed unilateral left necrotizing orchitis, with severe periorchitis and epididymitis. Similarly, necrotic testis mass encased within the markedly thickened tunics was previously recorded from *Brucella spp* infection [44,45]. Decreased fertility or sterility in bulls may develop according to the degree of necrosis [46]. Moreover, testicular enlargement was observed due to trauma as one of reproductive management of the ram [47]. 

The gross and microscopic genital lesions observed in this study, were in agreement with those described in the genital involvement in human salmonellosis [14,15,29,32,48]. Similar lesions have been described in sheep resulted from *S. arizona* infection [31]. On the contrary, firm and smaller testes associated with cryptorchidism were reported due to brucellosis in rams [21,23,31]. 

*Salmonella* could pass through M-cells overlying Peyer’s patches or through the epithelial lining of the lower part of small intestine or proximal colon to reach the sub epithelial location which is then transported to extra intestinal sites such as the liver, spleen and mesenteric lymph nodes [49].

The spleen is a lymphoid organ, in which old and damaged RBCs are phagocytized, iron is recycled, and where immune responses to blood-borne microorganisms are stimulated [50]. The characteristic splenic architecture comprises three major compartments: the white pulp (WP) where habitually B and T lymphocytes reside, the red pulp (RP), populated mainly by F4/80+ macrophages, and the margoinal zone (MZ), which separates the WP and RP and is populated by MOMA+ metallophilic macrophages [50,51]. Keeping this characteristic tissue architecture and outlines is critical for proper functioning of the spleen and for the initiation of immune responses against systemic diseases [50,51]. *Salmonella* has been reported to inhibit and suppress the host’s innate and adaptive immunity [52,53]. The most striking macroscopic finding observed was splenomegaly associated with a similar increase between red and white pulp ratio. Splenomegaly is supposed to result from plentiful increase in the numbers of phagocytes and lymphocytes [35]. Extensive thickening in the capsule was observed which could be due to fibrosis and diffuse inflammation. In accordance, severe thickening in the splenic arterial wall, cellular hyperplasia accompanied by wide patches of necrosis together with cellular depletion in the red and white pulp of the spleen were also represented in *Salmonella* infection [54]. Splenomegaly could be resulting from considerable intensity in immature RBC precursors and F4/80+ macrophages that are essential for the recycling of Hemi-associated iron [35]. Increase alkaline phosphatase accompanied with hemorrhage and hemosiderosis may be due to the fact that *Salmonella* could persist inside splenic macrophages, where extreme quantities of extracellular bacteria were propagated after the early acute stage of the infection [27].

## 4. Materials and Methods 

### 4.1. Ethical Approval

The procedures used in this work were approved by the National Animal Care and Use Committee of the Faculty of Veterinary Medicine, Assiut University, Egypt. All methods were performed in accordance with the relevant guidelines and regulations.

### 4.2. Sampling

The spleen, left testicle and epididymis were sent to the Animal Hygiene Lab and Histology Diagnostic Lab (Faculty of Veterinary Medicine, Assiut University) for microbiological and histopathological examinations, respectively.

### 4.3. Microbiological Examination

Ten grams of each infected tissue sample were incised using sharp sterilized scalpel and forceps and placed into a sterile homogenizer flask containing 45 mL of buffered peptone water. One mL of each homogenized sample was added to 9 mL of nutrient broth (Oxoid, Manchester U.K.), mixed and incubated (aerobically) at 37 °C overnight. A loopful from the broth was streaked on blood agar and incubated at 37 °C for 24 hrs. The isolates were characterized by gram staining (Gm -ve) and biochemical screening including TSI (red slope alkaline and yellow acid butt without H2S production), Simmon’s citrate (blue color) and Urease tests (yellow color) [55]. Colonies were purified on nutrient agar medium and subjected to VITEK 2-compact [56]. 

#### 4.3.1. VITEK Identification 

Bacterial suspensions were prepared by suspending the purified colonies in 3 ml of sterile saline in a polystyrene test tube. Turbidity was adjusted between 0.5 and 0.62 using DensiCHEKTM. Colonies were identified using the ID cards (GN (REF) 21341). All cards and their corresponding tubes were placed into the cassette and were transferred to the VITEK for filling and loading the cards to get the results report [56]. 

#### 4.3.2. PCR and Virulence Genes Identification 

PCR was performed and virulence genes were identified according to the Reference Lab for Veterinary Quality Control on Poultry Production, Animal Health research institute, Giza, Egypt. DNA was extracted from the overnight bacterial cultures using Patho Gene DNA/RNA extraction kit (iNtRON Biotechnology) according to the manufacturer’s instructions. Oligonucleotide primers used were supplied by Metabion (Germany) and are listed in Table 1 [57,58,59,60,61].

Primers were utilized in a 25 µL reaction containing 12.5 µL of EmeraldAmp Max PCR Master Mix (Takara, Japan), 1 µL of each primer of 20 pmol concentrations, 5.5 µL of water, and 5 µL of DNA template. The reaction was performed in an Applied Biosystems 2720 thermal cycler.

The PCR products were separated by electrophoresis on 1.5% agarose gel (Applichem, Germany, GmbH) in 1x TBE buffer at room temperature using gradients of 5 V/cm. For gel analysis, 20 µL of the products were loaded in each gel slot. A generuler 100 bp ladder (Fermentas, Germany) was used to determine the fragment sizes. The gel was photographed by a gel documentation system (Alpha Innotech, Biometra). 

#### 4.3.3. Gene Sequencing

Gene sequencing took place in the Molecular Biology Research Unit ISO/IEC (17025.2017), Assiut University.

Details for amplification of the universal primer (16S rRNA 27F and 1492R) oligonucleotide sequence used for the study were listed in Table 1. PCR products were purified using QIA quick PCR purification kit (Qiagen) and the sequencing reaction was performed using Bigdye terminator sequencing kit (Applied Biosystems) by sanger technique [62], then loaded on the sequencer instrument (ABI prism 310 genetic analyzer, Applied Biosystems). BLASTN searches were done using the NCBI server (http://www.ncbi.nlm.nih.gov/blast/Blast.cgi).

### 4.4. Histopathological Examination

#### 4.4.1. Gross Examination

Samples were examined for the presence of any induration, lesions or changes in the tissues architectures then dissected by cross-sectional cut to examine the abnormalities in the entire tissue.

#### 4.4.2. Light Microscopy

Each sample was divided into two parts. The first group was dissected (1 × 1 × 1 cm), and was fixed in Bouin’s fluid for 24 hours and processed according to description by Yosuf et al. [63]. Representative paraffin sections were stained by Harris haematoxylin and Eosin and Crossmon’s trichrome, Mallory triple trichrome. However, the second group was used for semithin sections.

##### Histochemical Investigation

The representative sections from the testicle, epididymis and spleen were stained with Pearls Prussian. The Prussian blue stain for iron demonstrates hemosiderin (blue granules) and brings out the green color of bile and the golden brown color of lipofuscin pigment. Lipofuscin pigments were stained with Long Zheil Nielsen.

##### Enzyme Histochemistry

Representatives sections were stained by the Gomori calcium method for alkaline phosphatase activity.

##### Semi Thin Section Preparations

Small specimens 2.0–3.0 mm long were taken from the testicle, epididymis and spleen, and then were used for semi thin sections fixed overnight in Karnovsky fixative [64], at 4 °C. Processing was done according to description by Abdel-Hakeem et al. [65]. The semi thin sections (1 μm) were cut using an ultra-microtome (Ultra Cut E, Reichert-Leica, Germany) and stained with toluidine blue. Suvarana et al. [66], cited all staining methods in Bancroft’s theory and practice of histological techniques.

All slides were examined using light microscopy following proper processing and staining. LeitzDialux 20 Microscope was used to examine the stained sections and photos were taken using a Canon digital camera (Canon power shot A95).

## 5. Conclusions

The study describes a case of unilateral epididymo-orchitis in a Holstein bull together with the isolation of *S.* Typhimurium and identification of its virulence genes (*stn, avr*A and *sop*B) in order to highlight this organism’s unusual clinical presentation. In the absence of pathogens causing orchitis, *S.* Typhimurium should be included in the differential diagnosis of bovine testicular and epididymal lesions afterward. Therefore, the possibility of *salmonella* infection at atypical sites should not be averted especially in the developing countries where *Salmonella* is endemic. Detection of *Salmonella* in feed, environment or animal is considered a serious public health problem and all measurements should be taken to interfere with its spreading. Infected individuals or herds should be isolated and assessed with treatment plan. Procedures to improve the biosecurity of the farm are necessary to exterminate the infection, including sanitation and disinfection, elimination of the carriers, chronically infected animals or birds, preventing manure contamination to feed, avoiding excessive antibiotics that alter gut microbiome leading to gene resistance development and alternatively boosting animal immunity through natural prebiotics. To ensure the withdrawal of infection, two consecutive whole-herd samplings with negative results are necessitated to consider a herd free from infection. 

## Figures and Tables

**Table 1:** Primers sequences, target genes, amplicon sizes and cycling conditions.

Target Gene	Primers Sequences	Amplified Segment (bp)	Primary Denaturation	Amplification (35 cycles)			Final Extension	Reference
				Secondary Denaturation	Annealing	Extension		
**16S rRNA**	27F (AGAGTTTGATCCTGGCTCAG)	1500	95 °C 5 min	95 °C 60 s	50 °C 60 s	72 °C 90 s	72 °C 10 min	Lane 1991
	1492R (GGTTACCTTGTTACGACTT)							
***Stn***	F(TTG TGT CGC TAT CAC TGG CAA CC)	617	94°C 5 min.	94°C 30 s	59°C 40 s	72°C 45 s	72 °C 10 min.	Murugkar et al., 2003
	R(ATT CGT AAC CCG CTC TCG TCC)							
***sopB***	F(TCAGAAGRCGTCTAACCACTC)	517	94 °C 5 min.	94 °C 30 s	58 °C 40 s	72 °C 45 s	72 °C 10 min.	Huehn et al. 2010
	R(TAC CGTCCT CAT GCACACTC)							
***avrA***	F(CCT GTA TTG TTG AGC GTC TGG)	422	94 °C 5 min.	94 °C 30 s	58 °C 40 s	72 °C 50 s	72 °C 10 min.	
	R(AGA AGA GCT TCG TTG AAT GTC C)							
***S.* Typhimurium *STM4495***	F(GGT GGC AAG GGA ATG AA)	915	94 °C 5 min.	94 °C 30 s	50 °C 40 s	72 °C 50 s	72 °C 10 min.	Liu et al., 2012
	R(CGC AGC GTA AAG CAA CT)							
***S.* Enteritidis *sefA***	F(GCAGCGGTTACTATTGCAGC)	310	94 °C 5 min.	94 °C 30 s	52 °C 30 s	72 °C 30 s	72 °C 7 min	Akbarmehr et al., 2010
	R(TGTGACAGGGACATTTAGCG)							

## Supplementary Materials

The following are available online at https://www.mdpi.com/2076-0817/9/4/314/s1, text and Figure S1. (a and b) Bull showing unilateral testicular enlargement (arrows). c: morphology of left testis (arrows) showing the enlargement after slaughtering the animal. d: Markedly thickened fibrosis’ tunics (Line with double arrowheads) and scattered yellow patches of necrosis in the testicle (white arrows). Figure S2. a-b cut section in the enlarged testis and epididymis after fixation showing the greatly thickened tunics and scattered yellow patches (arrows) in the testis and epididymis. Figure S3 Photomicrographs of paraffin sections of the testis of infected bull a: showing thick organized fibrinous tunics (line with double arrowheads). Note: hemorrhage (stars), b and c: Scattered fibrinous sheets within the tunics (arrowheads). Blood vessels magnified in image d. d: Showing the wall of the blood vessels engorged with extensive hemorrhage and inflammatory cellular infiltration. e: Higher magnification of selected square showing the hemorrhage (double stars). f, and g: the tunica albuginea infiltrated with extensive fibrin threads (arrowheads), h: chronic inflammatory cells (1, selected square showing the giant cells, arrowhead, GC) and (2, selected square showing lymphocytes, arrowhead, Ly). i: Blood vessels are dilated (arrowheads) and surrounded by dense fibrous tissue. Note: infiltration of inflammatory cells in a magnified square. j: Granulomatous structures ranged from the micro to a large one (macro granuloma and micro granuloma, arrowheads). K: showing the granulomatous structure formed from a productive cellular reaction around coagulative central necrosis (**), surrounded by fibrosis (double arrowheads). a, c, e, f, g, and h: Sections stained by Hematoxylin and eosin. b, d, j, k: Sections stained by Mallory triple trichrome stain. i stained by Gomori calcium method for alkaline phosphatase activity. Figure S4. Photomicrographs of Paraffin sections of the testis of infected bull showing a-c: atrophied seminiferous tubules were damaged and a near-total absence of spermatogenic cells, (arrowheads). Note, b showing a higher magnification of the selected white square form a. d: Necrosis inside seminiferous tubules infiltrated with inflammatory cells (arrowheads). e-i: Granulomatous inflammatory lesions (arrowheads) showing different stages of granuloma formation, beginning with destructed seminiferous tubules bordered by fibrosis (double arrowheads) and inflammatory cells (star) and debris, to coagulative necrosis (j and k) of the seminiferous tubules with losing its the cellular structure surrounding with fibrosis and chronic cellular infiltration (double stars), till the formation of coalesced granulomatous masses (l, double arrowheads). a-f, h and i sections stained by Hematoxylin and eosin. g, j, k, l sections stained by Mallory triple trichrome. Figure S5. (a-d and f) photomicrographs of Paraffin sections and (e) semi thin section of the testis of infected bull showing a and b: the seminiferous epithelium (ST) become necrotic and desquamates with tubular destruction and per-tubular fibrosis (double arrowheads). The dense inflammatory infiltrates, predominantly in the interstitium (IT), (stars). c and d: higher magnification showing the seminiferous tubules is obliterated and replaced by numerous inflammatory cells, Lymphocytes (Ly), giant cells (GC, arrowhead), (macrophage, arrowhead) and debris. (e) Macrophages, with foamy acidophilic cytoplasm (arrowheads, macrophage) within the seminiferous epithelium. (f)Alkaline phosphatase reaction showing the reaction of inflammatory cells within the seminiferous tubules (ST, arrowhead) and in the interstitium (interstitium, arrowhead). a-d sections stained by Hematoxylin and eosin, e: section stained by toluidine blue, f: sections stained by the Gomori calcium method for alkaline phosphatase activity. (Figure S6): a, b, d, e and f photomicrographs of Semi thin sections stained with toluidine blue stain and c: paraffin section stained by Gomori calcium method for alkaline phosphatase activity of infected testis showing, atrophy, and necrosis of seminiferous tubules (ST, arrowhead), the tubular outline is retained in the affected area, but the seminiferous epithelium is destructed and the interstitial (IT) is highly infiltrated inflammatory cells mainly lymphocytes(Ly, arrowhead). c: Strong positive reaction in the inflammatory cells (double arrowheads). Note, macrophages (macrophage, arrowhead), plasma cell, arrowhead, destructed neutrophil (N, arrowheads). (Figure S7): a Low magnification, b, and d higher magnification photomicrographs of paraffin sections stained by the Gomori calcium method for alkaline phosphatase and c and d: Semi thin sections of infected testis stained with toluidine blue showing, the interstitial tissue (IT) was expanded by dense inflammation. The inflammatory infiltrate is comprised of lymphocytes (Ly, arrowheads), neutrophils (N), (mast cells), and macrophage cells (macrophage, arrowhead, M). Note the deposition of fibers in e (double arrowheads). **Figure S8**. a–c, e–h, j-o photomicrographs of paraffin sections and d, I, p-r semi thin sections of epididymis of infected bull. a, b, e, f, g, h, j-n; stained by hematoxylin and eosin. d, i, P–r stained by toluidine blue. O stained by Crossman’s trichrome stain. a: showing large multifocal to coalescing pyogranulomatous structures, losing normal tubular architecture (arrowheads). b: Higher magnification of selected white square showing marked thick tunics (line) due to fibrosis. c: Crossman’s trichrome stain showing fibrosis (arrowheads), sever hemorrhage underneath the capsule (white stars). d: Semi-thin section showing the fibrous tissue (arrows). e: Serosal layer is selected square (1) magnified in f and layer underneath the tunics in the selected square (2) magnified in g. f: the serosal layer with fibrous tissue (arrows) and Giant cells infiltration(arrow heads). g: Diffuse inflammatory cell infiltration (double arrowheads). h: Noticeable abundant fibroblast cells filled the serosa layer over capsular structure (arrowheads) ) and magnified square. i: Diffuse inflammatory cell infiltration mainly macrophages (macrophage, arrowheads). j: marked thick wall and fibrosis (arrowheads) with extensive hemorrhage (stars). K: blood vessels congested (arrowheads), l–o: granulomatous structure (Arrowheads), N, p–r: composed of neutrophil cells (N, arrowheads), macrophages (m, arrowheads) and surrounded by fibrous tissue (double arrowheads). (Figure S9): Photomicrographs of Paraffin sections of Epididymis of infected bull showing a: coalesced pyogranulomatous (double arrowheads).b and c: pyogranulomatous (*) was composed of abundant degenerate neutrophils and epithelioid macrophages (arrowheads) surrounding extensive areas of necrosis with mineralized cellular debris (**). The Selected area from b magnified in image c. d: interstitial tissue around the granulomatous structure was infiltrated with diffuse inflammatory cells. Note, giant cells magnified in the square (GC). e: diffuse inflammatory cells mainly lymphocytes (Ly, arrowheads), Note, congested blood capillaries (BC, *).f: higher magnification of selected square show macrophage (M, arrowheads), fibroblast (double arrowheads) cells in addition to telocytes (TC, arrowheads). a stained by Crossman’s trichrome, b-f are stained by hematoxylin and eosin. (Figure S10): Photomicrographs of Paraffin section a-c, g-i and semi-thin sections d-f of Epididymis of infected bull showing: a-c, marked diffuse hemorrhage (*), extravasated hemorrhage in the interstitial tissue (*), the blood vessels are engorged with blood (arrows). d-f, the blood vessels are thickened with fibrosis (arrowheads), Inflammatory cellular infiltration mainly macrophages (double arrowheads) and neutrophil (N, arrowheads). g-i, macrophage laden with golden yellow hemosiderin pigment (arrowheads). i, magnification of white selected square in h. Note, hemosiderin pigment (double arrowheads). (double arrowheads). a and g stained by hematoxylin and Eosin stain, b, and c by Crossman’s stain, d-f stained by toluidine blue, h and i stained zheil Nielsen stain. (Figure S11): a and b, Spleen of infected bull showing enlarged, firm and nodular splenic surface (arrows). (Figure S12): The Spleen of infected bull showing large nodular masses (granulomatous structures) extended from the splenic surface until the middle of splenic parenchyma. (arrowhead). Splenic surface covered by marked thickened fibrous connective tissue (double arrowheads) in some areas than other parts (arrowhead). (Figure S13): a-d, g-i paraffin sections and e-f semi-thin sections of spleen of infected bull showing a-c, segmented thick and moderately normally thickened splenic capsule (Line 1, 2 and arrows). d and e, the blood vessel was dilated and engorged with blood (double arrowheads). Note, the marked thickened areas showed severe fibrosis (arrows), f, Focal areas of capsule showed vacuolization due to it was invaded by vacuolar macrophages (arrowheads). g, Micro granulomatous reactions (**) in the splenic capsule were seen. Note, selected Black Square was magnified in the next image. h, inflammatory cellular infiltration principally at serous membrane over the capsule, eosinophil’s (white arrows), epithelioid macrophage(M) and giant cells (GC, arrowheads). i, the different staining intensity of alkaline phosphatase activity in the inflammatory cells (arrowheads, arrows). j, the alkaline phosphatase activity of lymphocytes in the micro granulomatous reaction (Ly, white arrows) and enlarged in White square. k, Magnified black square from image j showing the alkaline phosphatase activity in lymphocytes infiltration principally at serous membrane over the capsule (Ly, arrowheads).l, the alkaline phosphatase activity of epithelioid macrophage (m, arrows).a, b, d stained by hematoxylin and eosin, c, g, h stained by Crossomon’s trichrome, i-l stained by the Gomori calcium method for alkaline phosphatase activity. e and f stained by toluidine blue stain. (Figure S14): a-j paraffin sections and k semi thin section of spleen of infected bull showing a stages of granuloma formation from micro-granuloma to the well-developed one (arrows). b-f, some granulomatous reactions formed from central coagulative necrosis surrounded by inflammatory cells (white arrows) and (i-j, arrows) the growing one formed from a circumscribed collection of chronic inflammatory cells mainly vacuolated epithelioid macrophages and neutrophil cells, g and h, blood vessels are dilated and engorged with blood (arrows) and surrounded by intensive fibrous reaction (k, arrows).a, b, d, f, g and j stained by Gomori calcium method for alkaline phosphatase activity. c and e stained by hematoxylin and eosin, h and i stained by Crossomon’s trichrome, k stained by toluidine blue. (Figure S15): a-d and f paraffin sections and e, semi-thin sections of spleen showing marked fibrosis (arrows) in interstitial and around the white pulp is observed (double arrowheads). b and c, depletion of lymphocytes in some areas that was characterized by reducing densities of lymphocytes (arrowheads). d, Hemorrhage (*) and existing of erythrocytes around blood vessels (BV). e, mainly neutrophils(N), lymphocytes (Ly, arrows) and macrophage filled with pigment (macrophage, arrowheads) and mast cells especially around lymphoid follicles were seen. f diffuse inflammatory cellular infiltration, (arrows), in addition to, presence of modified thick-walled blood vessels (BV, line with double arrowheads).a-d and f stained by Crossomon’s trichrome, e stained by toluidine blue. (Figure S16): a-e paraffin sections and f-h semi-thin sections of spleen showing: a-d, Hemorrhage (*, RBCS) with red blood cells extravasated the cellular structures, leukocytes infiltration (double arrowheads). Note, golden yellow lipofuscin pigments in a. Note the thickening of septa (S) in b. In image c, fibrosis around the arterial wall (arrows). e, the splenic artery was constricted with protrusion of the endothelial cell (EC, arrowheads) lining toward the lumen in addition to the marked hyperplasia to the arterial wall and fibrosis around it (arrows), with leukocytes infiltration (double arrowheads). f low magnification of splenic artery and g higher magnification of selected square showing vacuolization in the focal area around splenic artery (SA). Note, epithelioid cellular infiltration (arrows). h, interstitial cellular infiltration including, Neutrophil (N, double arrows heads) and lymphocytic (Ly, white arrows) and macrophage (M).a stained by hematoxylin and eosin, b-e stained by Crossomon’s trichrome, f-h stained by toluidine blue. (Figure S17): paraffin sections stained by the Gomori calcium method for alkaline phosphatase activity showing: a, lymphocytes stained positively (arrows). b destructed neutrophil and macrophage stained positively (arrows). c and d, low and higher magnification of splenic trabecula were thickened due to swelled and distended smooth muscular trabecula. Blood vessel inside the splenic trabecula was dilated and engorged with blood. Note, Diffuse lymphocytes (arrowheads) and neutrophils (arrows) stained positively with alkaline phosphatase. e, Strong positive staining of alkaline phosphatase in the macrophage (arrows) within the red pulp. f, the reaction of alkaline phosphatase in macrophage within the interstitial space (square2).Note, the reaction in lymphocytes (square 1) and neutrophil in (square 3) within the blood vessels. (Figure. S18): a, c, e-h paraffin sections and b and d, semi-thin sections of spleen showing: a, marked diffuse hemorrhage, the blood vessels are severely dilated and engorged with blood, b-d extravasated hemorrhage in the interstitial tissue (*). Note, N is neutrophil, (macrophage, arrowheads), (RBCs, arrows) and orange lipofuscin pigment(arrowheads), arrows pointed to leukocyte infiltration. In d showing the macrophage with lipofuscin pigment.e Low and f, high magnification of selected red square showing the diffuse hemosiderosis spots (arrowheads) of dispersed bluish precipitations of iron pigments in the white and red pulp of the spleen. g, golden brown Lipofuscin pigment(black arrows) stained positively with Long zheil Nielsen’s, green staining of granules within the macrophage. h, Macrophage with green stained granules. a and c stained by HX and eosin, b and d stained by toluidine blue, e and f stained by Prussian blue stain for iron. g stained with Long zheilNielsen; h stained with Crossmon ‘s trichrome stain.
