# Supplementary material for: Salmonella Typhimurium Triggered Unilateral Epididymo-Orchitis and Splenomegaly in a Holstein Bull in Assiut, Egypt: A Case Report
Source: Pathogens. 2020 Apr 24;9(4):314. doi: 10.3390/pathogens9040314 (PMC7238186; doi:10.3390/pathogens9040314)
Supplement: Supplementary file 1 [file pathogens-09-00314-s001.pdf]

Testis and epididymis

Necrotic intratubular pyogranulomatus orchitis and epididymitis

Macroscopically Findings:

Supplementary images showed the observed effect in this report was unilateral scrotum swells and dough due to massively inflammatory alterations in the tunics—Supplementary Figure S1a, b, the necrotic parenchyma became softened by liquefaction and the organ came to be like a pus-filled cavity enclosed by a dense layer of a connective-tissue capsule—Supplementary Figure S1c, d. On the cut section, there were dispersed yellow foci of necrosis that coalesced and gave off total testicular necrosis—Supplementary Figure S2a, b. The epididymis had a soft consistency due to pyogranuloma formation and the accumulation of fibrinopurulent exudate supplementary Figure S2a, b.

Microscopically

Additional details in supplementary images as the followings: In general, the histopathological finding is ultimately in the form of severe necrosis surrounded by fibrosis and inflammatory cells, in addition to lymphocytic infiltration in the interstitial and degeneration of seminiferous tubules. Histopathological examination of the testes revealed that the inflammatory envelopment of the tunics is analogous to that which occurs at any serous membrane with organization producing adhesions between the parietal and visceral layers. The testicular capsule showed marked thickened tunica vaginals and tunica albuginea due to the severe fibrosis—Supplementary Figure S3 a, b and hemorrhage underneath the tunics—Supplementary Figure S3 d, e, in addition to the presence of scattered fibrinous sheets within the tunics—Supplementary Figure S3 b, c. Blood vessels were dilated and engorged with blood—Supplementary Figure S3 d, e and Supplementary Figure S3k. The vessels were surrounded by diffuse inflammatory cellular infiltration (Figure S3i). Extensive chronic inflammatory cellular infiltration—Supplementary Figure S3f, g, h resulted in salmonella granuloma formation ranging from micro to large ones—Supplementary Figure S3 j. Inside the testes, the seminiferous tubular epithelium of tubules and the interstitial tissues turn out to be necrotic and desquamates—Supplementary Figure S4a, d, e. The tubular outline was kept in the affected area, but the seminiferous epithelium was destroyed—Supplementary Figure S4b,c , Supplementary Figure S5 a and b, and interchanged by numerous macrophages and multinucleated giant cells which circumscribed neutrophils and debris—Supplementary Figure S4d,e and Supplementary Figure S5c,d,e. In some areas, there were granulomatous inflammations (Salmonella granulomas) with obliteration of seminiferous tubules Supplementary Figure S4f,g,h,i. Multifocal to coalescing areas of granulomatous inflammation enclosed and separated the seminiferous tubules—Supplementary Figure S4l. .Centre local circumscribed districts of inflammatory foci encompassed plentiful granular to flocculent eosinophilic cellular debris interspersed with abundant pyknotic nuclear debris and karyorrhectic remnants. These areas were surrounded by a zone containing predominantly large polygonal cells (epithelioid macrophages) admixed with lymphocytes and neutrophils—Supplementary Figure S5c,d,e,f and Supplementary Figure S6a–f. Modified vascularization was often evident as small, plump, endothelium-lined blood vessels arranged perpendicular to the inflammatory foci and containing few RBCs—Supplementary Figure S4 j, k. The remaining seminiferous tubules within and adjacent to areas of inflammation, lacked evidence of spermatocyte production or contained degenerating cells—Supplementary Figure S6d,e,f. Sever inflammatory cellular reaction composed from diffuse neutrophils, lymphocytes, macrophages and mast cells infiltrated the interstitial tissue between the seminiferous tubules—Supplementary Figure S7a–e.

The epididymis showed marked thick tunics with organized fibrosis and diffuse inflammatory cellular infiltration—Supplementary Figure S8a, b, c—sever hemorrhage underneath the tunics, in addition to the diffuse hemorrhage in the interstitial tissue—Supplementary Figure S8a,c and

Supplementary Figure S8j, k. Presence of small and large-sized granulomatous structures, at the epididymal capsule—Supplementary Figure S8b ,m, n, o, i composed mainly from aggregated neutrophils and macrophage cells—Supplementary Figure S8 p, q, r and Supplementary Figure S10 f .

The serosal layer with fibrous tissue and Giant cells infiltration. The layer underneath the tunics with diffuse inflammatory cell infiltration mainly macrophages and noticeable abundant fibroblast cells filled the serosa layer over capsular structure Supplementary Figure S8 d-i

The epididymal tissue revealed large multifocal to coalescing pyogranulomas effacing normal tubular architecture—Supplementary Figure S9a, b. The pyogranulomas were comprised of abundant degenerate neutrophils and epithelioid macrophages surrounding extensive areas of necrosis—Supplementary Figure S9c–f. The histopathological findings showed chronic granulomatous inflammatory reaction inside the epididymal intertubular fibrous connective tissue, which separates and surrounds the tubules—Supplementary Figure S9b, c, f. In this case, the quantity of fibrinous exudates was significant. There is frequently focal necrotizing epididymitis complicated with diffuse hemorrhage—Supplementary Figure S10c, g, h, i. The blood vessels were constricted, thickened with fibrosis—Supplementary Figure S10a, b, d, e.

Spleen (Splenomegaly)

Macroscopic findings:

Splenomegaly, the spleen weight was enlarged and increased in weight, two times larger than normal—Supplementary Figure S11a,b. The splenic surface has a firm and large whitish nodular structure resulting from granulomatous reaction, which makes the spleen rigid, in addition to the marked thickening of the connective capsule surrounding its surface—Supplementary Figure S12a, b.

Microscopically

There were marked inflammations in the spleen. Noticeable thickening of connective capsule and trabeculae inter-bracketed which was also moderately normal thickened—Supplementary Figure S13a, b, c. The thick segments of the capsule had numerous chronic granulomatous reactions, which render the spleen rigid and nodular—Supplementary Figure S13g. However, diffuse connective tissue filled the capsular structure; in some areas, it had vacuolar appearance Supplementary Figure S13e, f. There was diffuse neutrophilic cell infiltration, in addition to the presence of multinucleated giant cells, especially in the splenic capsular structure—Supplementary Figure S13h, j, k, i, l. Blood vessels were engorged with blood especially under the serosa layer in addition to the extravagated blood filtrated the under-serosa layer of splenic capsule—Supplementary Figure S13c,d,h. Inside the spleen, there was excessive inflammatory cellular infiltration, particularly around lymphoid follicles as observed in Supplementary Figure S14g, h. Different stages of granulomatous reaction were observed beginning from coagulative necrosis to the complete well-developed granuloma, which was composed mainly from epithelioid macrophage and neutrophils in addition to lymphocytic cells infiltration—Supplementary Figure S 14a–f. Under high magnification, vacuolated macrophage cells were seen to represent the almost content of the granuloma—Supplementary Figure S14 i, j, k .The granulomatous reactions were formed principally around the white pulp, while some micro and well-formed granulomas were observed in the red pulp—Supplementary Figure S14 a, b, c, d. The splenic artery was constricted with protrusion of the endothelial cell lining in the direction of the lumen—Supplementary Figure S15d and Supplementary Figure S 16b, c, d, e. severe thickening in the splenic arterial wall due to cellular hyperplasia is shown in Supplementary Figure S15f. There was a diffuse chronic inflammatory cellular infiltration around the splenic artery, chiefly vacuolated macrophage, neutrophil, lymphocytes and mast cells, those in addition to red blood cell extravasation around the splenic artery areas—Supplementary Figure S15e. Some focal areas in the spleen had exhaustion of lymphocytes, which was characterized by reducing concentrations of lymphocytes—Supplementary Figure S15 a, b, c. Splenic trabeculae were

significantly thickened due to swelled and distended smooth muscular trabeculae in some areas—Supplementary Figure S17c, d. Diffuse epithelioid macrophages and neutrophils were diffusely filtrated the spleen and stained positively with alkaline phosphatase were clearly observed in Supplementary Figure S17a, b, e, f. hemorrhage and hemosiderosis were noticed. These spots of haemosidrosis were stained positively with Prussian blue stain and could be seen as diffuse and dispersed bluish precipitations of iron pigments in white and red pulp of spleen—Supplementary Figure S18a–h.

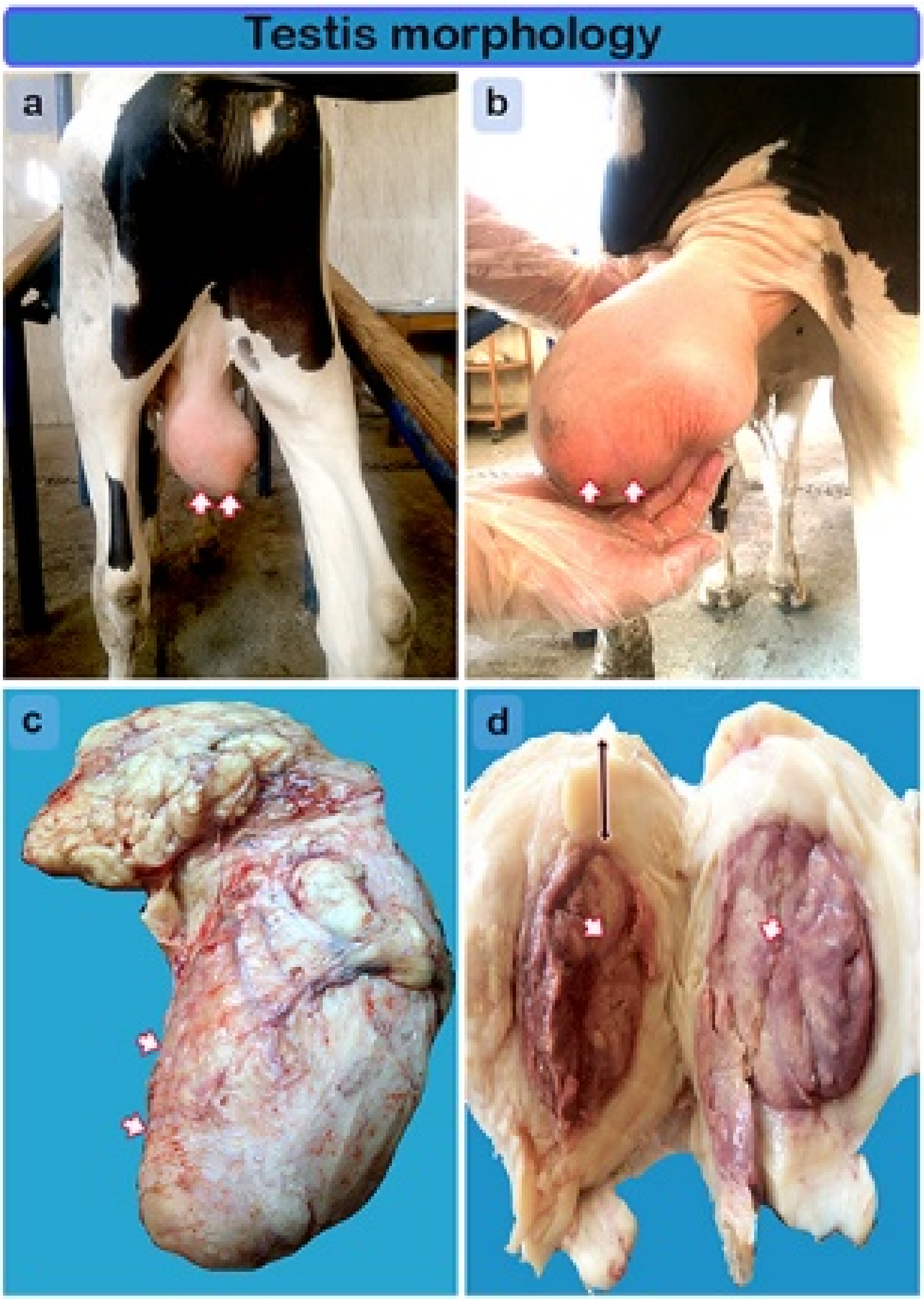

Figure S1. (a and b): Bull showing unilateral testicular enlargement (arrows). c: Morphology of left testis (arrows) showing the enlargement after slaughtering the animal. d: Markedly thickened fibrosis' tunics (line with double arrowheads) and scattered yellow patches of necrosis in the testicle (white arrows).

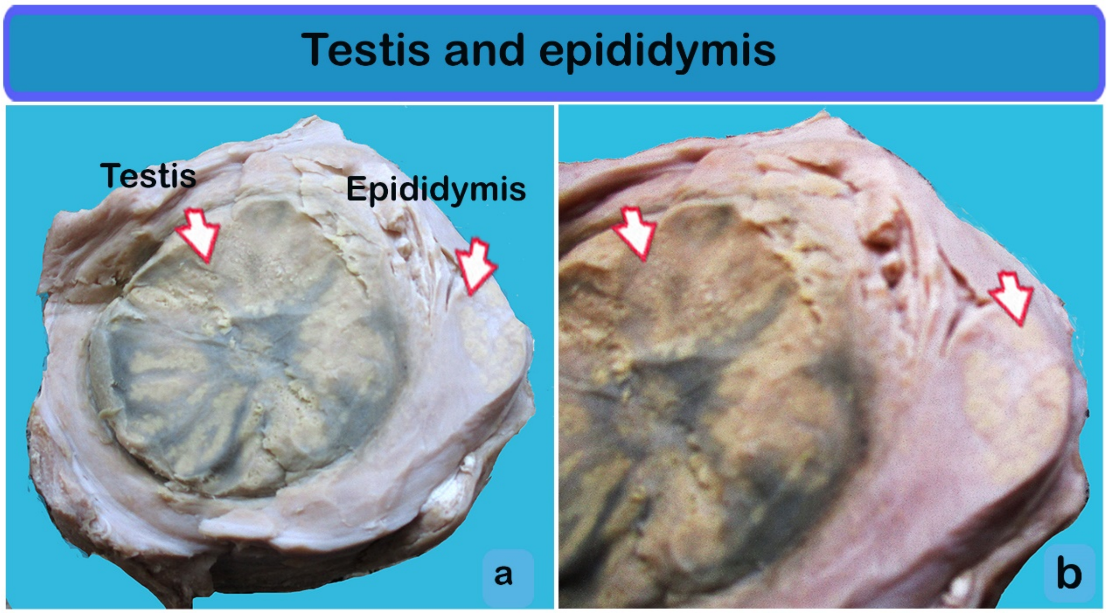

Figure S2. a–b cut section in the enlarged testis and epididymis after fixation showing the greatly thickened tunics and scattered yellow patches (arrows) in the testis and epididymis.

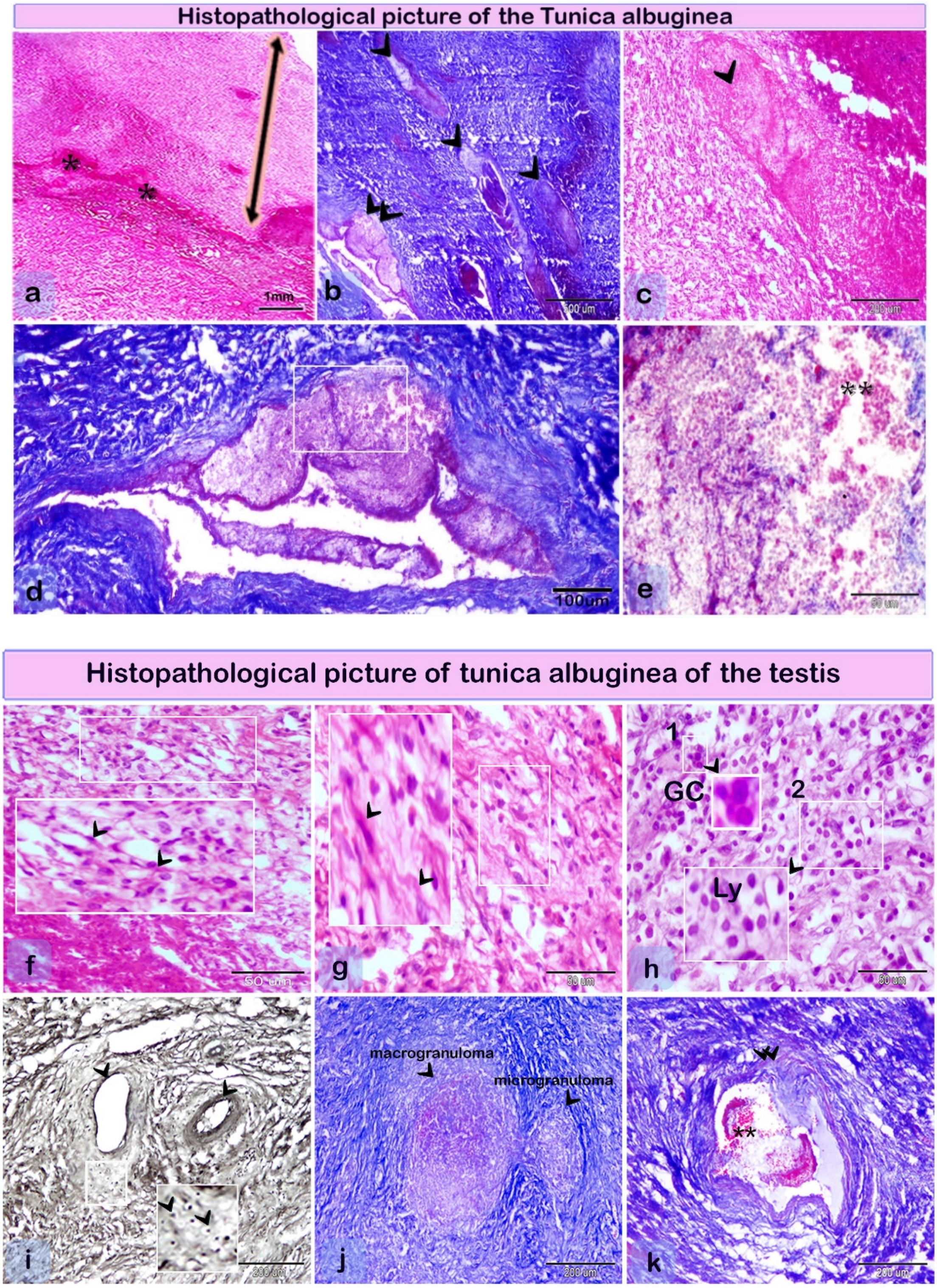

Figure S3. Photomicrographs of paraffin sections of the testis of infected bull.

a: showing thick organized fibrinous tunics (line with double arrowheads). Note: hemorrhage (stars), b and c: Scattered fibrinous sheets within the tunics (arrowheads). Blood vessels magnified in image d. d: Showing the wall of the blood vessels engorged with extensive hemorrhage and inflammatory cellular infiltration. e: Higher magnification of selected square showing the hemorrhage (double stars). f, and g: the tunica albuginea infiltrated with extensive fibrin threads (arrowheads), h: chronic inflammatory cells (1, selected square showing the giant cells, arrowhead, GC) and (2, selected square showing lymphocytes, arrowhead, Ly). i: Blood vessels are dilated (arrowheads) and surrounded by

dense fibrous tissue. Note: infiltration of inflammatory cells in a magnified square. j: Granulomatous structures ranged from the micro to a large one (macro granuloma and micro granuloma, arrowheads). K: showing the granulomatous structure formed from a productive cellular reaction around coagulative central necrosis (**), surrounded by fibrosis (double arrowheads). a, c, e, **f, g, and h:** Sections stained by Hematoxylin and eosin. b, d, **j, k**: Sections stained by Mallory triple trichrome stain. **i stained by Gomori calcium method for alkaline phosphatase activity**

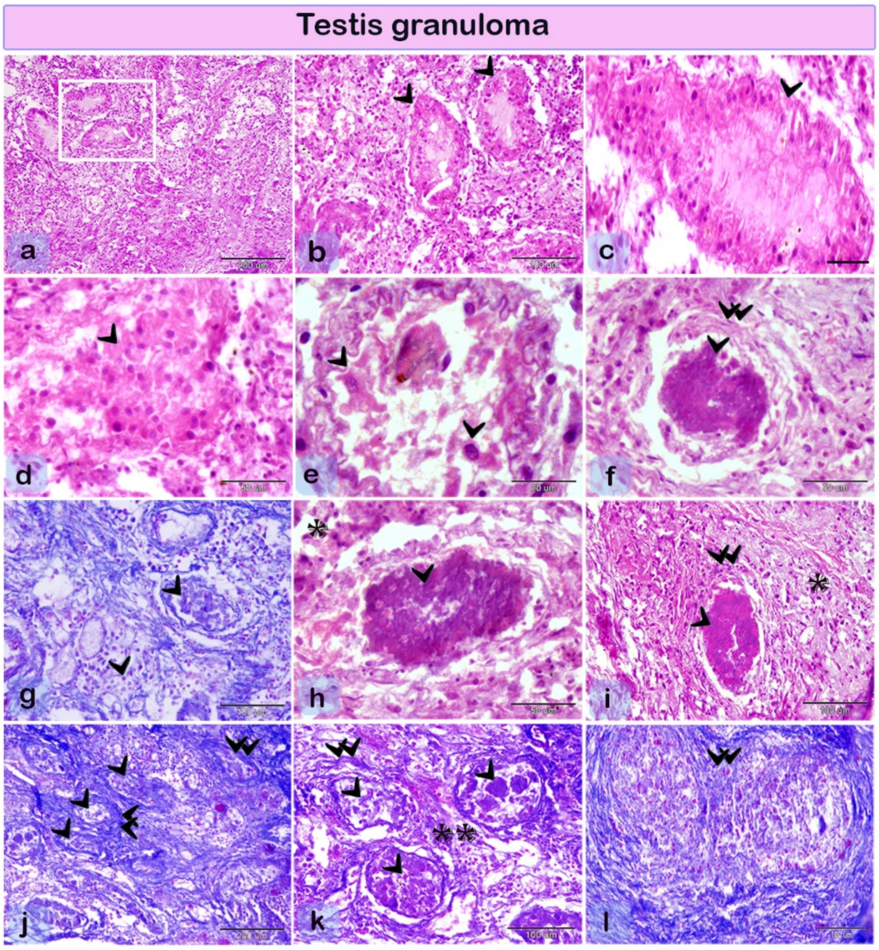

Figure S4. Photomicrographs of Paraffin sections of the testis of infected bull showing a–c: atrophied seminiferous tubules were damaged and a near-total absence of spermatogenic cells (arrowheads). Note, b showing a higher magnification of the selected white square form a. d: Necrosis inside seminiferous tubules infiltrated with inflammatory cells (arrowheads). e–i: Granulomatous inflammatory lesions (arrowheads) showing different stages of granuloma formation, beginning with destructed seminiferous tubules bordered by fibrosis (double arrowheads) and inflammatory cells (star) and debris, to coagulative necrosis (j and k) of the seminiferous tubules with losing its the cellular structure surrounding with fibrosis and chronic cellular infiltration (double stars), till the formation of coalesced granulomatous masses (l, double arrowheads). a–f, h and i: sections stained by Hematoxylin and eosin. g, j, k, l: sections stained by Mallory triple trichrome.

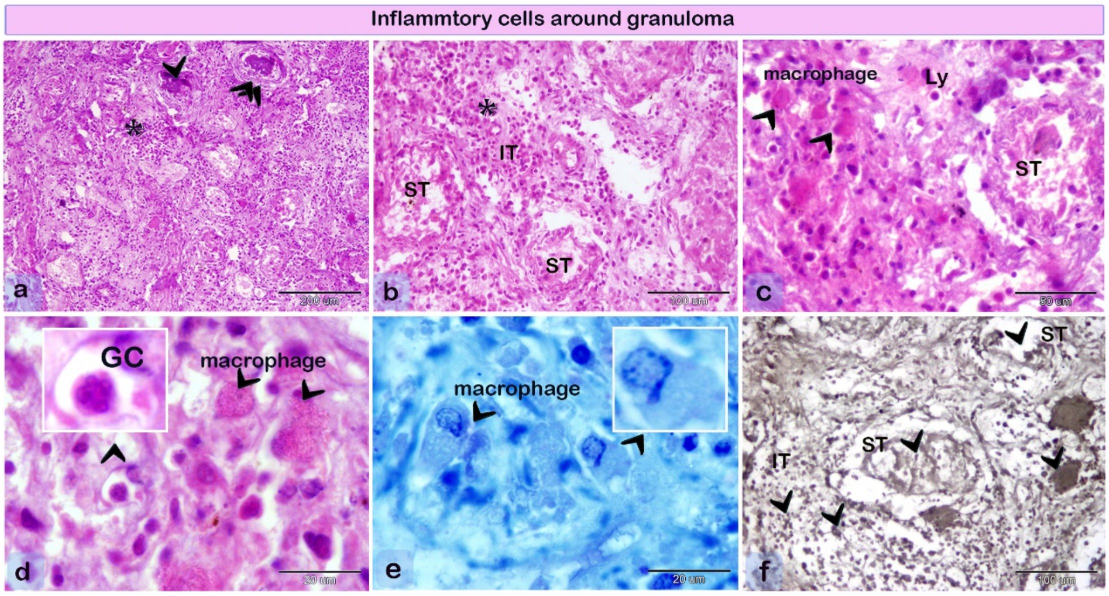

Figure S5. (a–d and f) photomicrographs of Paraffin sections and (e) semi thin section of the testis of infected bull showing a and b: the seminiferous epithelium (ST) become necrotic and desquamates with tubular destruction and per-tubular fibrosis (double arrowheads). The dense inflammatory infiltrates, predominantly in the interstitium (IT), (stars). c and d: higher magnification showing the seminiferous tubules is obliterated and replaced by numerous inflammatory cells, Lymphocytes (Ly), giant cells (GC, arrowhead), (macrophage, arrowhead) and debris. (e) Macrophages, with foamy acidophilic cytoplasm (arrowheads, macrophage) within the seminiferous epithelium. (f) Alkaline phosphatase reaction showing the reaction of inflammatory cells within the seminiferous tubules (ST, arrowhead) and in the interstitium (interstitium, arrowhead). a–d sections stained by Hematoxylin and eosin, e: section stained by toluidine blue, f: sections stained by the Gomori calcium method for alkaline phosphatase activity.

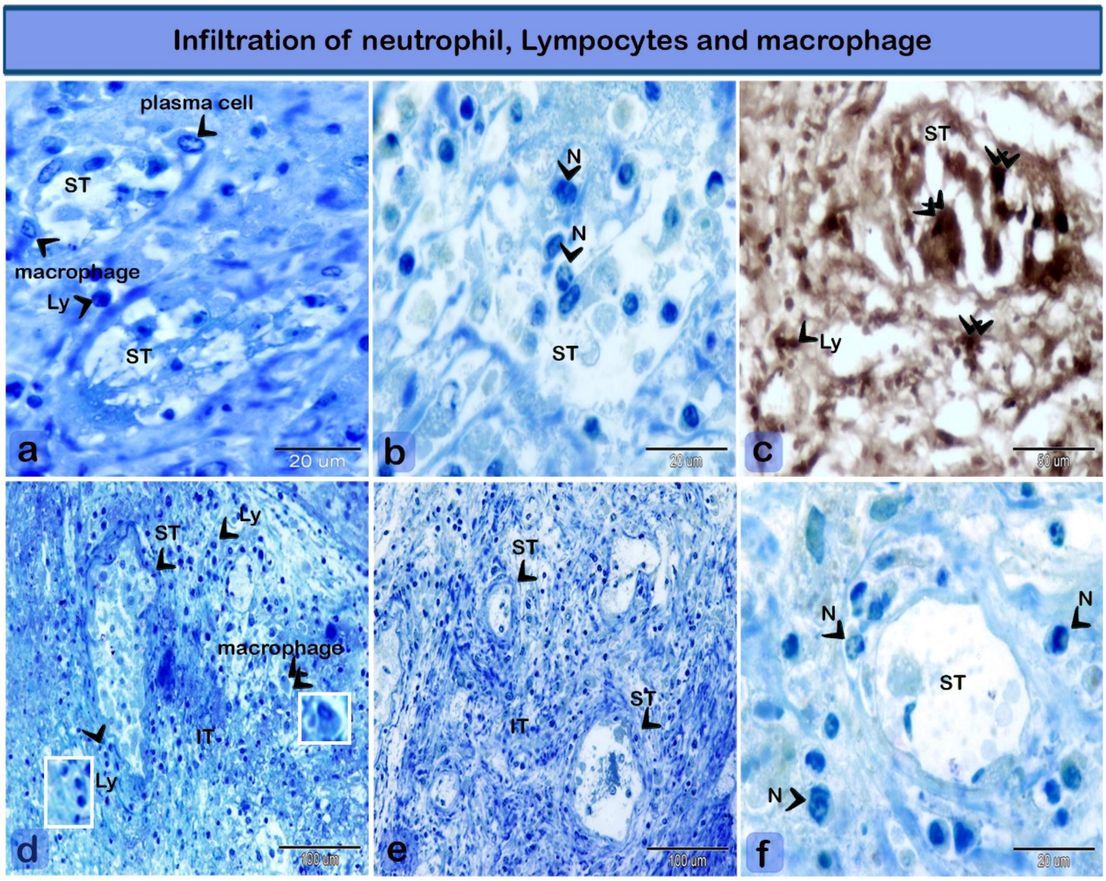

Figure S6. a, b, d, e and f: photomicrographs of semi-thin sections stained with toluidine blue stain and c: paraffin section stained by Gomori calcium method for alkaline phosphatase activity of infected testis showing, atrophy, and necrosis of seminiferous tubules (ST, arrowhead), the tubular outline is retained in the affected area, but the seminiferous epithelium is destructed and the interstitial (IT) is highly infiltrated inflammatory cells mainly lymphocytes(Ly, arrowhead). c: strong positive reaction in the inflammatory cells (double arrowheads). Note: macrophages (macrophage, arrowhead), plasma cell, arrowhead, destructed neutrophil (N, arrowheads).

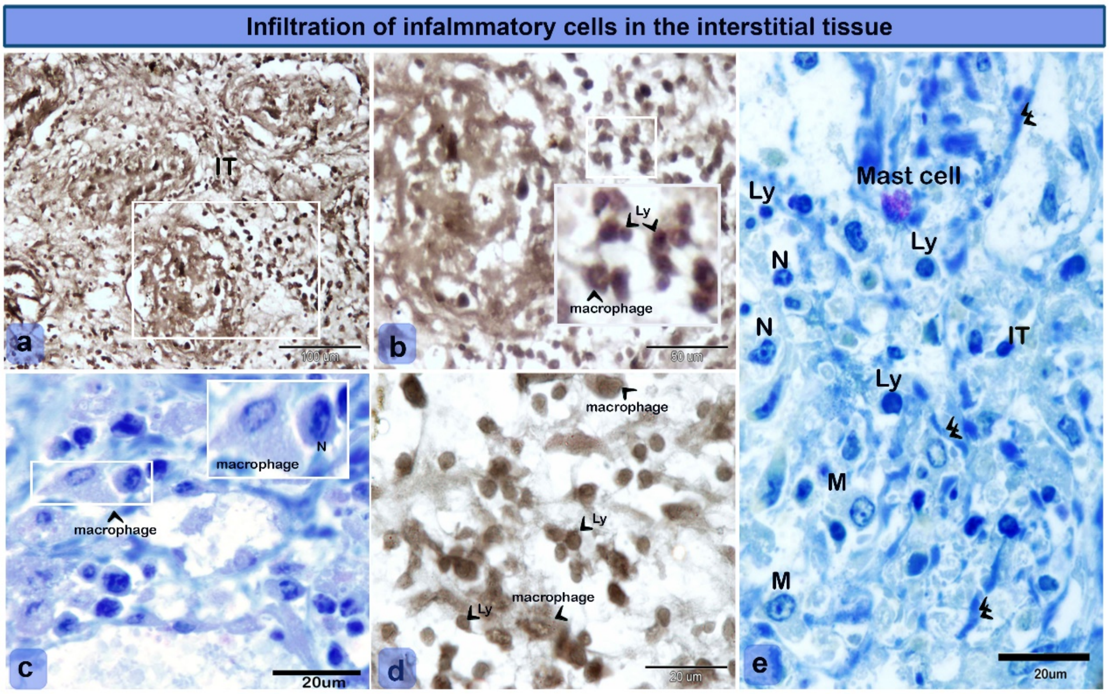

Figure S7. a: Low magnification; b and d: higher magnification photomicrographs of paraffin sections stained by the Gomori calcium method for alkaline phosphatase and; c and d: semi-thin sections of infected testis stained with toluidine blue showing, the interstitial tissue (IT) was expanded by dense inflammation. The inflammatory infiltrate is comprised of lymphocytes (Ly, arrowheads), neutrophils (N), (mast cells), and macrophage cells (macrophage, arrowhead, M). Note the deposition of fibers in e (double arrowheads).

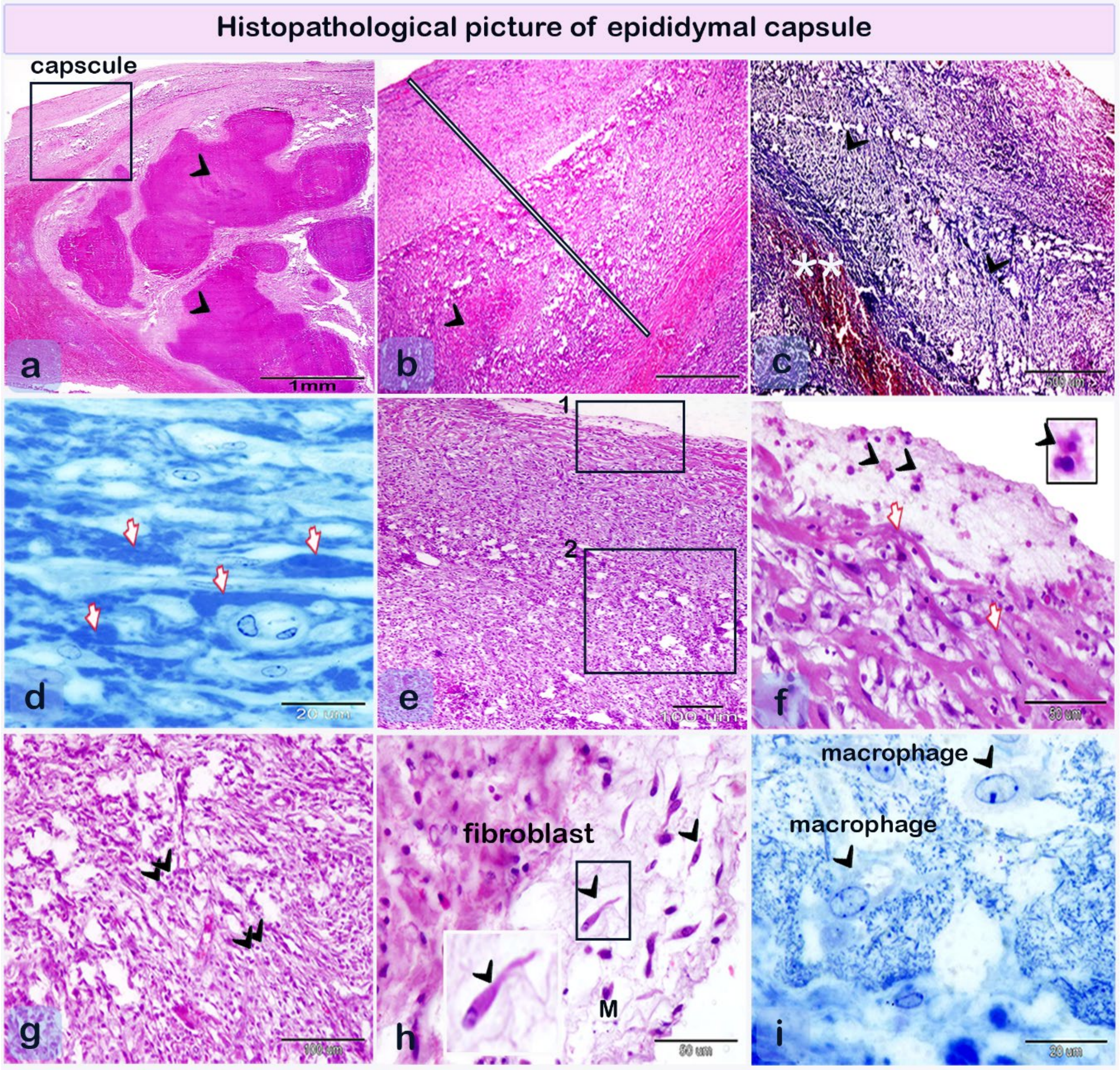

Histopathological picture of epididymal capsule
capscule
a
1mm
b
c
d
e
1
2
f
g
fibroblast
M
h
macrophage
macrophage
i

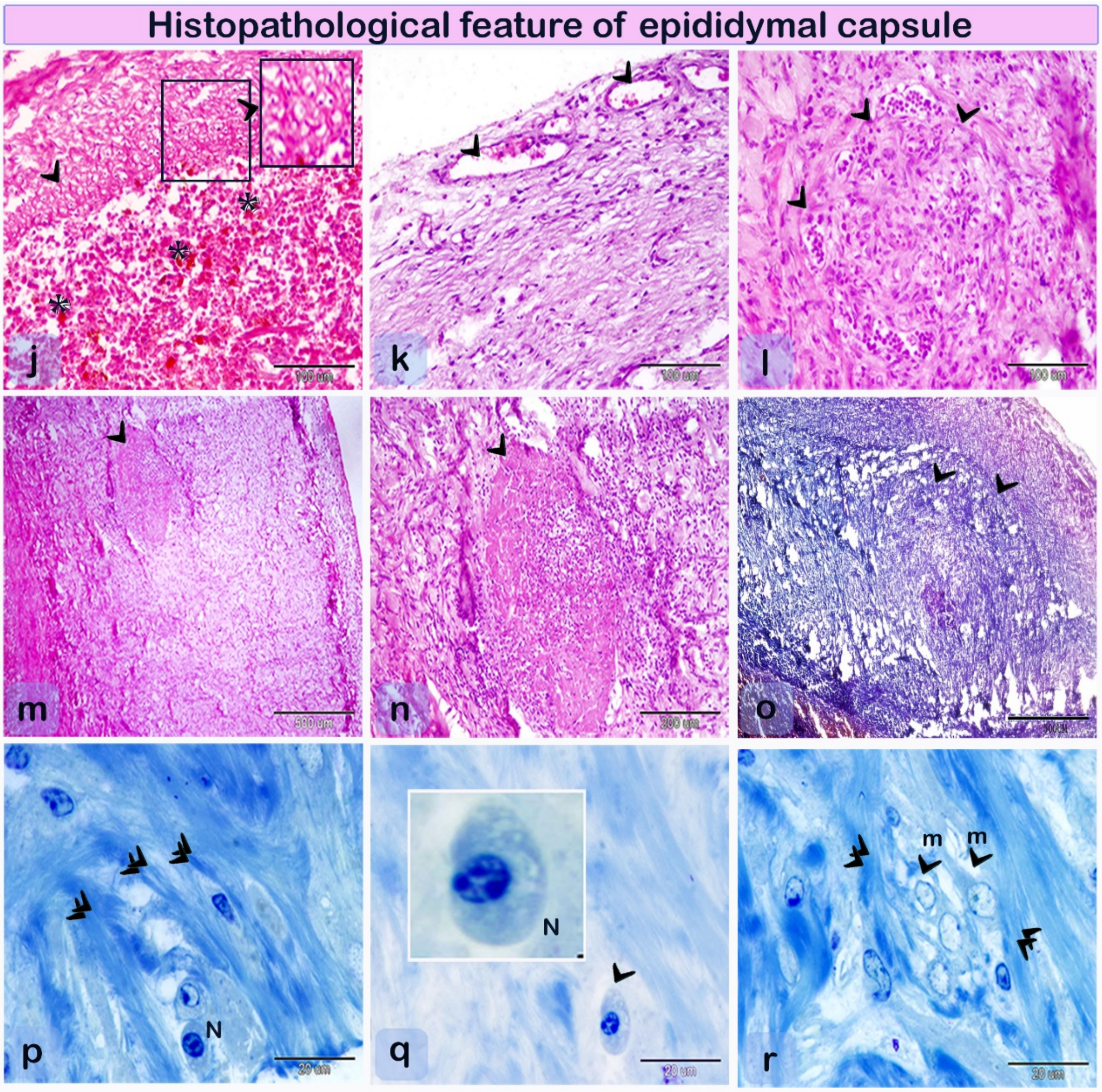

Figure S8. a–c, e–h j-o photomicrographs of paraffin sections and d, I, p-r semi thin sections of epididymis of infected bull .a, b, e, f, g, h, j-n; stained by hematoxylin and eosin. d, i, P–r stained by toluidine blue. O stained by Crossman's trichrome stain.

a: showing large multifocal to coalescing pyogranulomatous structures, losing normal tubular architecture (arrowheads). b: Higher magnification of selected white square showing marked thick tunics (line) due to fibrosis. c: Crossman's trichrome stain showing fibrosis (arrowheads), sever hemorrhage underneath the capsule (white stars). d: Semi-thin section showing the fibrous tissue (arrows). e: Serosal layer is selected square (1) magnified in f and layer underneath the tunics in the selected square (2) magnified in g. f: the serosal layer with fibrous tissue (arrows) and Giant cells infiltration(arrow heads). g: Diffuse inflammatory cell infiltration (double arrowheads). h: Noticeable abundant fibroblast cells filled the serosa layer over capsular structure (arrowheads) and magnified square. i: Diffuse inflammatory cell infiltration mainly macrophages (macrophage, arrowheads). j: marked thick wall and fibrosis (arrowheads) with extensive hemorrhage (stars). K: blood vessels congested (arrowheads), l–o: granulomatous structure (Arrowheads), N, p–r: composed of neutrophil cells (N, arrowheads), macrophages (m, arrowheads) and surrounded by fibrous tissue (double arrowheads.

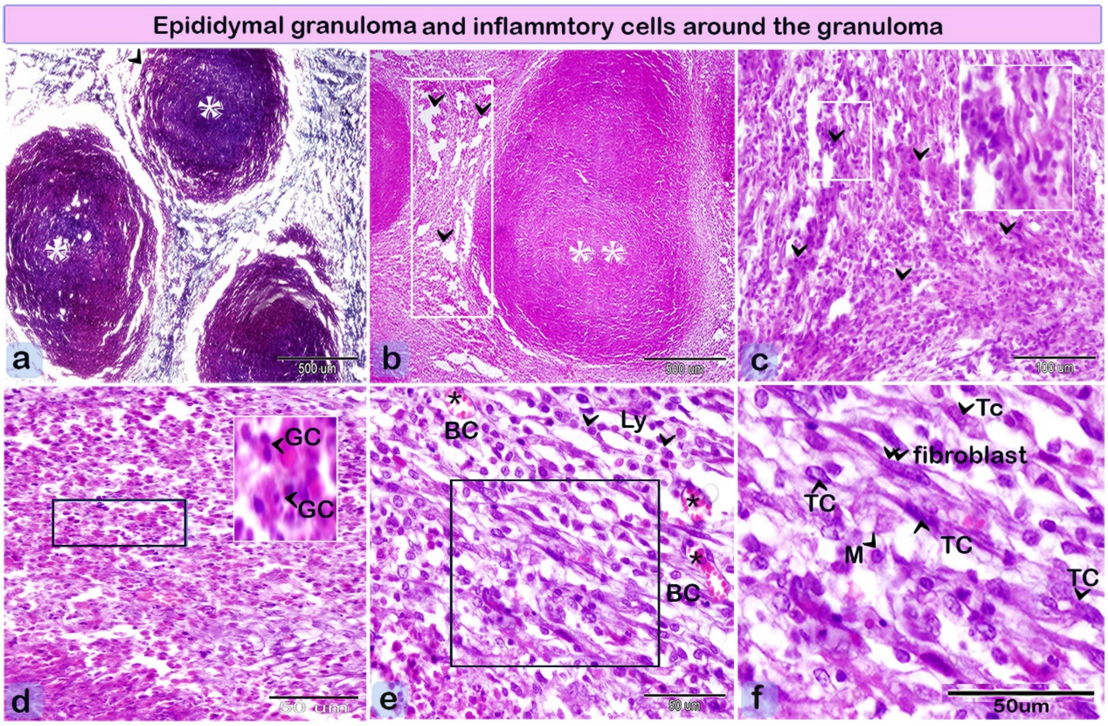

Figure S9. Photomicrographs of Paraffin sections of epididymis of infected bull showing a: coalesced pyogranulomatous (double arrowheads). b and c: pyogranulomatous (*) was composed of abundant degenerate neutrophils and epithelioid macrophages (arrowheads) surrounding extensive areas of necrosis with mineralized cellular debris (**). The selected area from b magnified in image c. d: interstitial tissue around the granulomatous structure was infiltrated with diffuse inflammatory cells. Note: giant cells magnified in the square (GC). e: Diffuse inflammatory cells mainly lymphocytes (Ly, arrowheads). Note: congested blood capillaries (BC, *). f: Higher magnification of selected square show macrophage (M, arrowheads), fibroblast (double arrowheads) cells in addition to telocytes (TC , arrowheads). a: stained by Crossman's trichrome, b–f are stained by hematoxylin and eosin.

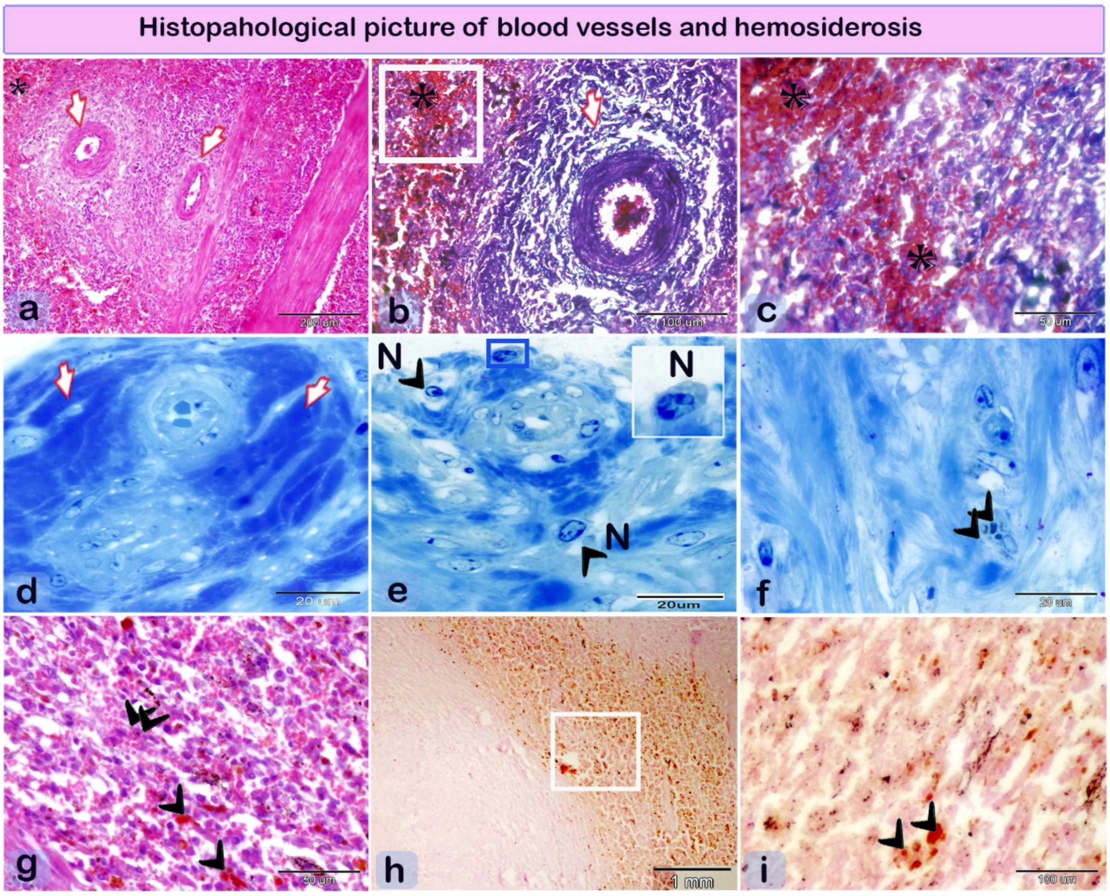

Figure S10. Photomicrographs of Paraffin section a–c, g–i and semi-thin sections d–f of epididymis of infected bull showing: a–c, marked diffuse hemorrhage (*), extravasated hemorrhage in the interstitial tissue (*), the blood vessels are engorged with blood (arrows). d–f, the blood vessels are thickened with fibrosis (arrowheads), inflammatory cellular infiltration mainly macrophages (double arrowheads) and neutrophil (N, arrowheads). g–i, macrophage laden with golden yellow hemosiderin pigment (arrowheads). i: Magnification of white selected square in h. Note: hemosiderin pigment (double arrowheads). a and g: stained by hematoxylin and eosin stain, b, and c by Crossman's stain, d–f stained by toluidine blue, h and i stained zheil Nielsen stain.

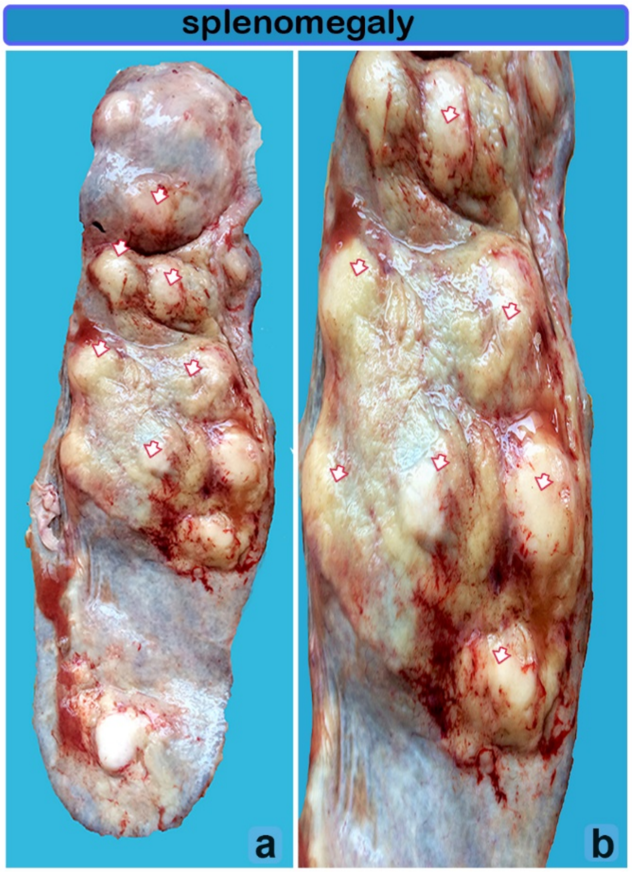

Figure S11. a and b: Spleen of infected bull showing enlarged, firm and nodular splenic surface (arrows).

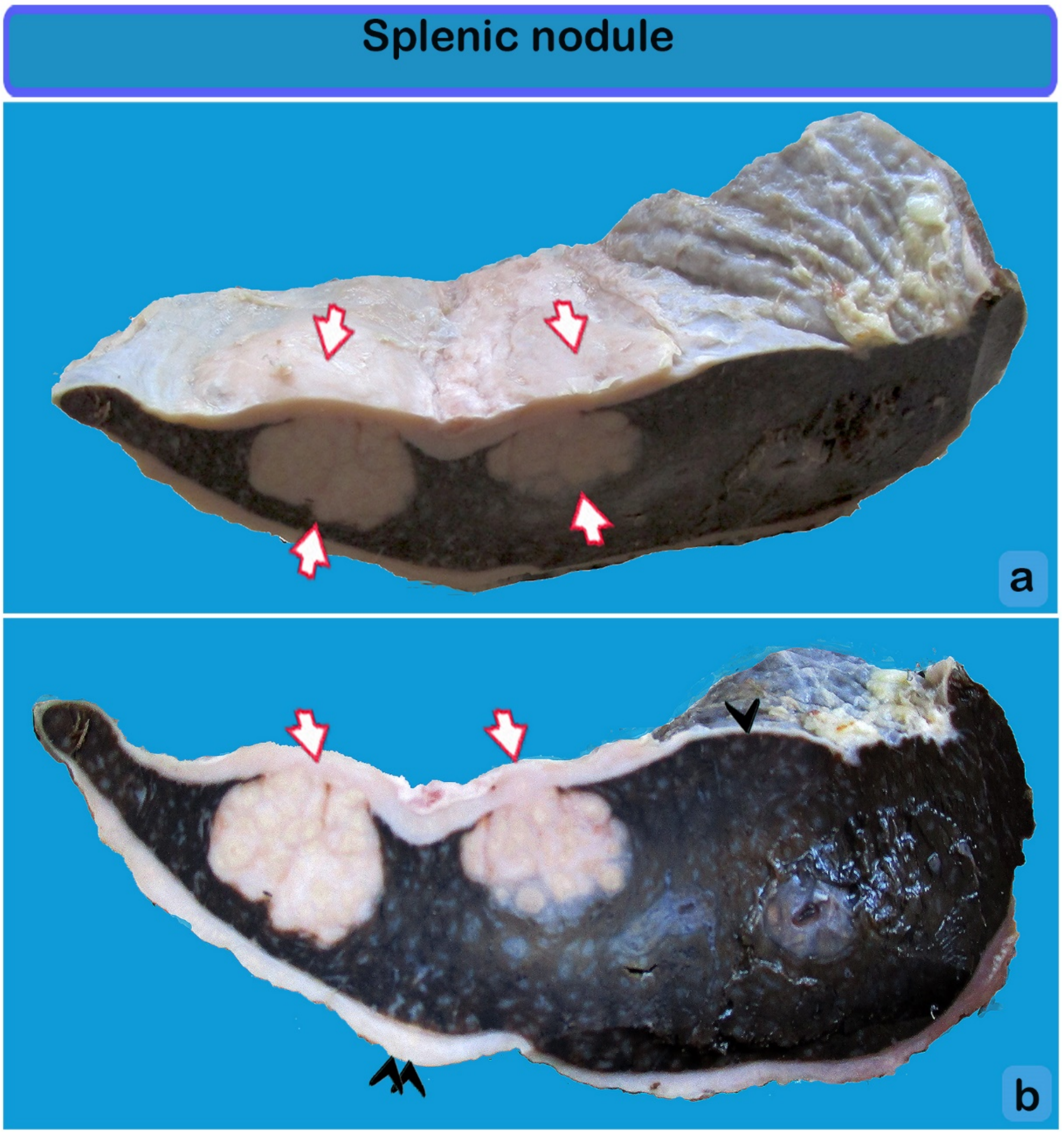

Figure S12. The spleen of infected bull showing large nodular masses (granulomatous structures) extended from the splenic surface until the middle of splenic parenchyma. (arrowhead). Splenic surface covered by marked thickened fibrous connective tissue (double arrowheads) in some areas than other parts (arrowhead).

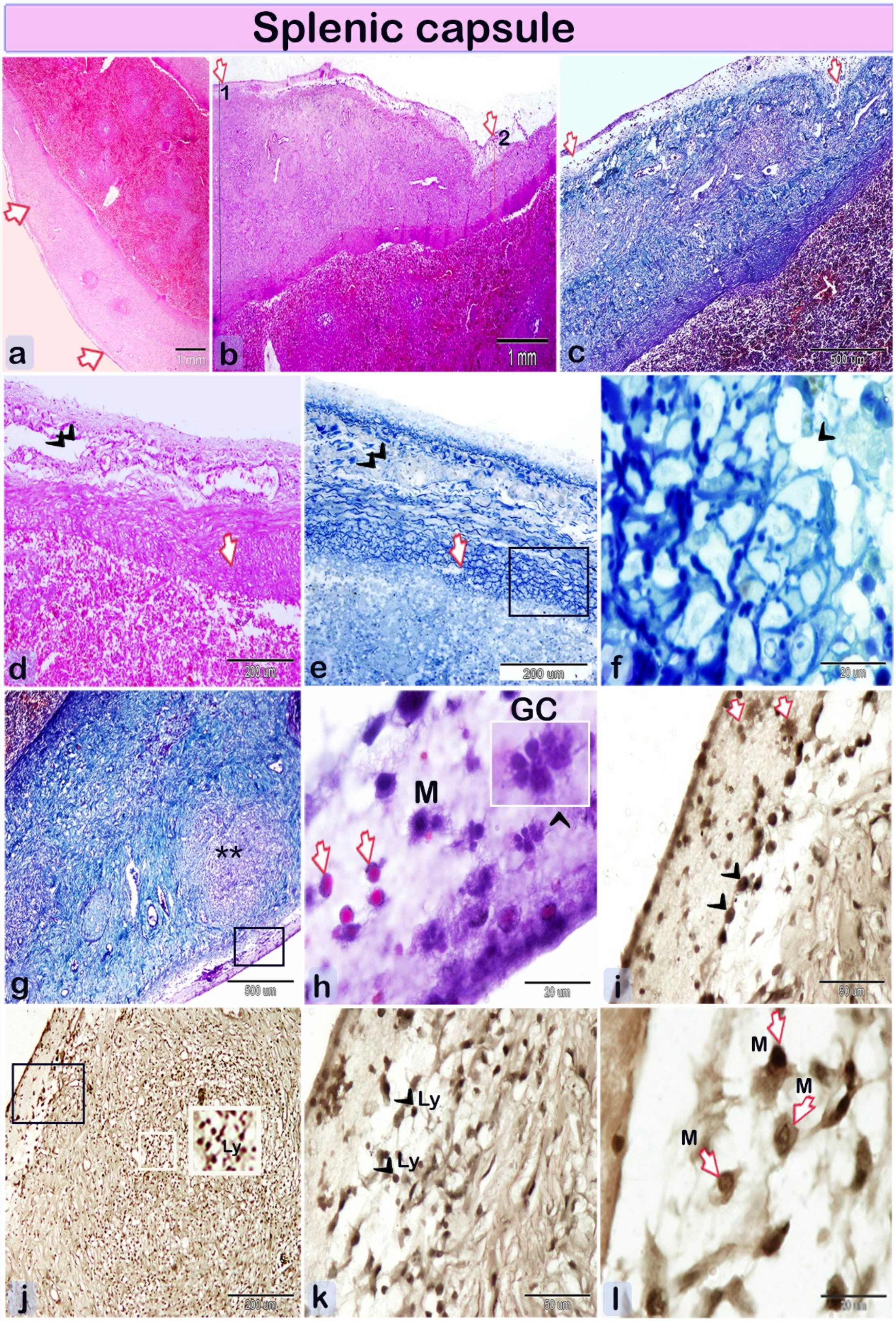

Figure S13. a–d, g–i paraffin sections and e-f semi-thin sections of spleen of infected bull showing a–c, segmented thick and moderately normally thickened splenic capsule (line 1, 2 and arrows). d and e, the blood vessel was dilated and engorged with blood (double arrowheads). Note: the marked thickened areas showed severe fibrosis (arrows), f: focal areas of capsule showed vacuolization due to it was invaded by vacuolar macrophages (arrowheads). g: Micro granulomatous reactions (**) in the splenic capsule were seen. Note: selected black square was magnified in the next image. h: inflammatory cellular infiltration principally at serous membrane over the capsule, eosinophil's

(white arrows), epithelioid macrophage (M) and giant cells (GC, arrowheads). i: the different staining intensity of alkaline phosphatase activity in the inflammatory cells (arrowheads, arrows). j: the alkaline phosphatase activity of lymphocytes in the micro granulomatous reaction (Ly, white arrows) and enlarged in White square. k: magnified black square from image j showing the alkaline phosphatase activity in lymphocytes infiltration principally at serous membrane over the capsule (Ly, arrowheads). l) The alkaline phosphatase activity of epithelioid macrophage (m, arrows). a, b, d: stained by hematoxylin and eosin; c, g, h: stained by Crossomon's trichrome; i–l: stained by the Gomori calcium method for alkaline phosphatase activity. e and f: stained by toluidine blue stain.

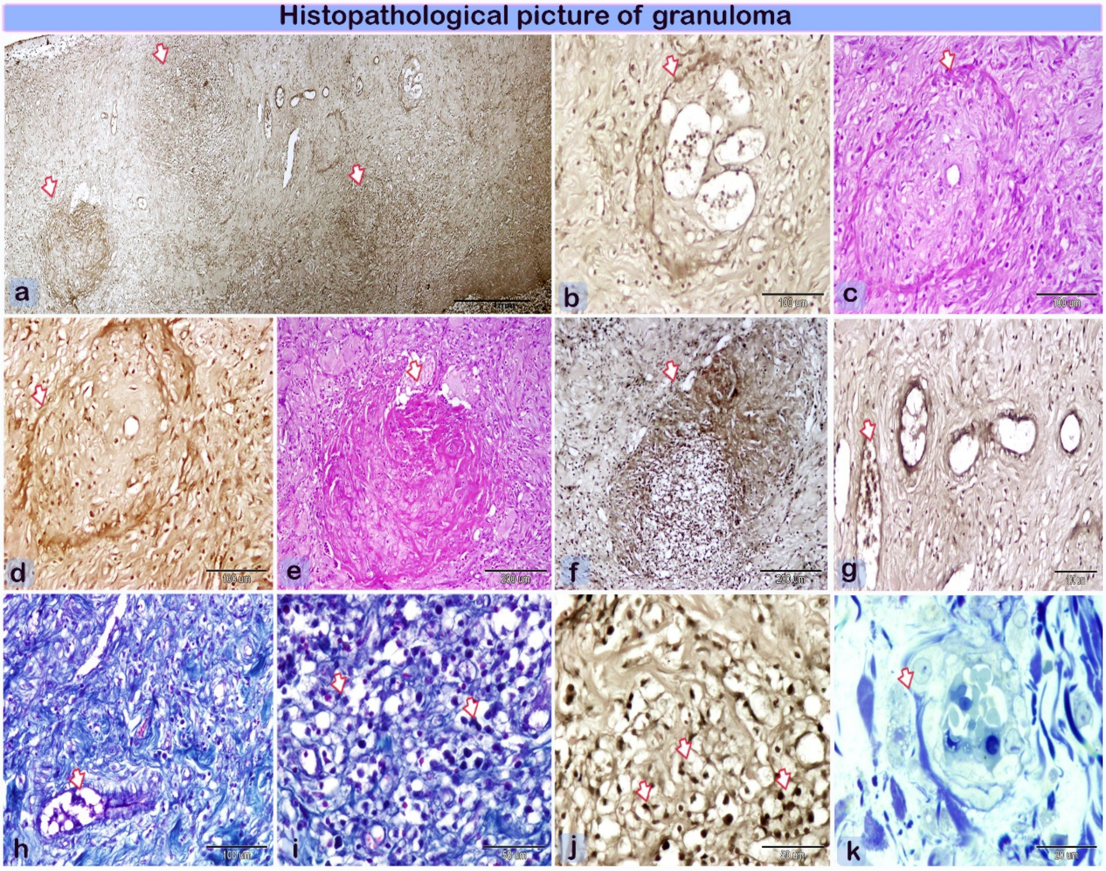

Figure S14. a–j paraffin sections and k semi thin section of spleen of infected bull showing a stages of granuloma formation from micro-granuloma to the well-developed one (arrows). b–f, some granulomatous reactions formed from central coagulative necrosis surrounded by inflammatory cells (white arrows) and (i–j, arrows) the growing one formed from a circumscribed collection of chronic inflammatory cells mainly vacuolated epithelioid macrophages and neutrophil cells, g and h, blood vessels are dilated and engorged with blood (arrows) and surrounded by intensive fibrous reaction (k, arrows). a, b, d, f, g and j: stained by Gomori calcium method for alkaline phosphatase activity. c and e: stained by hematoxylin and eosin, h and i: stained by Crossomon's trichrome, k stained by toluidine blue.

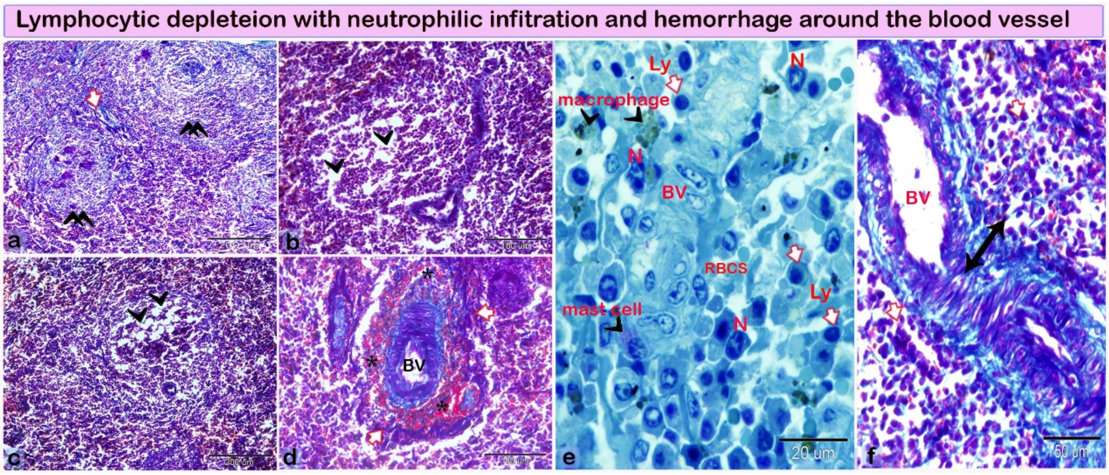

Figure S15. a–d and f paraffin sections and e, semi-thin sections of spleen showing marked fibrosis (arrows) in interstitial and around the white pulp is observed (double arrowheads). b and c, depletion of lymphocytes in some areas which was characterized by reducing densities of lymphocytes (arrowheads). d, Hemorrhage (*) and existing of erythrocytes around blood vessels (BV). e, mainly neutrophils (N), lymphocytes (Ly, arrows) and macrophage filled with pigment (macrophage, arrowheads) and mast cells especially around lymphoid follicles were seen. f: diffuse inflammatory cellular infiltration, (arrows), in addition to, presence of modified thick-walled blood vessels (BV, line with double arrowheads). a–d and f stained by Crossomon's trichrome, e stained by toluidine blue.

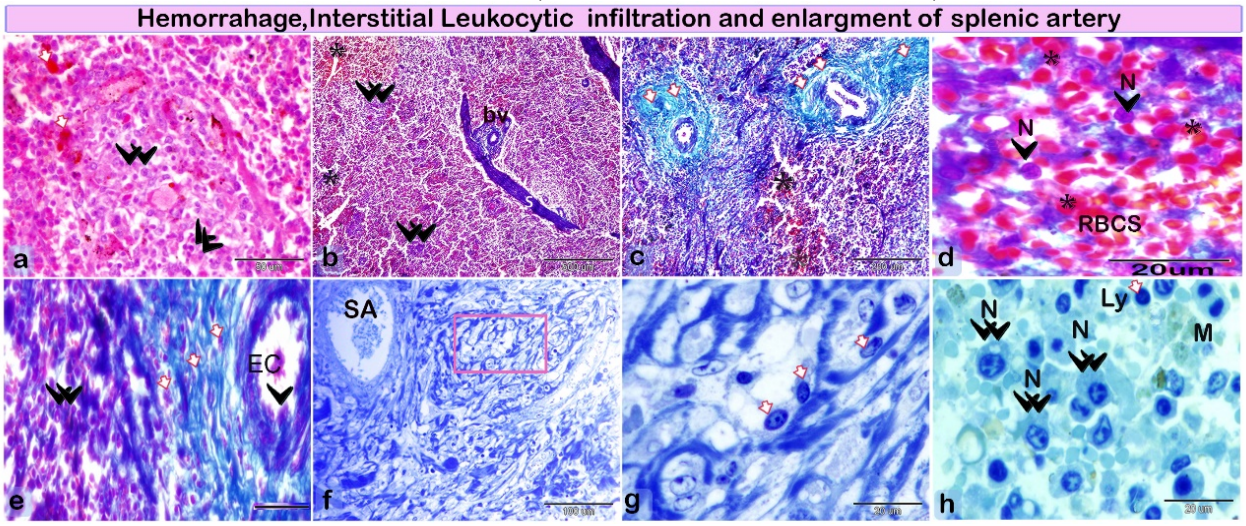

Figure S16. a–e paraffin sections and f–h semi-thin sections of spleen showing: a–d, hemorrhage (*, RBCS) with red blood cells extravasated the cellular structures, leukocytes infiltration (double arrowheads). Note: golden yellow lipofuscin pigments in a. Note the thickening of septa (S) in b. In image c: fibrosis around the arterial wall (arrows). e: The splenic artery was constricted with protrusion of the endothelial cell (EC, arrowheads) lining toward the lumen in addition to the marked hyperplasia to the arterial wall and fibrosis around it (arrows), with leukocytes infiltration (double arrowheads). f: low magnification of splenic artery and g higher magnification of selected square showing vacuolization in the focal area around splenic artery (SA). Note, epithelioid cellular infiltration (arrows). h: Interstitial cellular infiltration including, neutrophil (N, double arrows heads) and lymphocytic (Ly, white arrows) and macrophage (M). a: Stained by hematoxylin and eosin, b–e: stained by Crossomon's trichrome, f–h stained by toluidine blue.

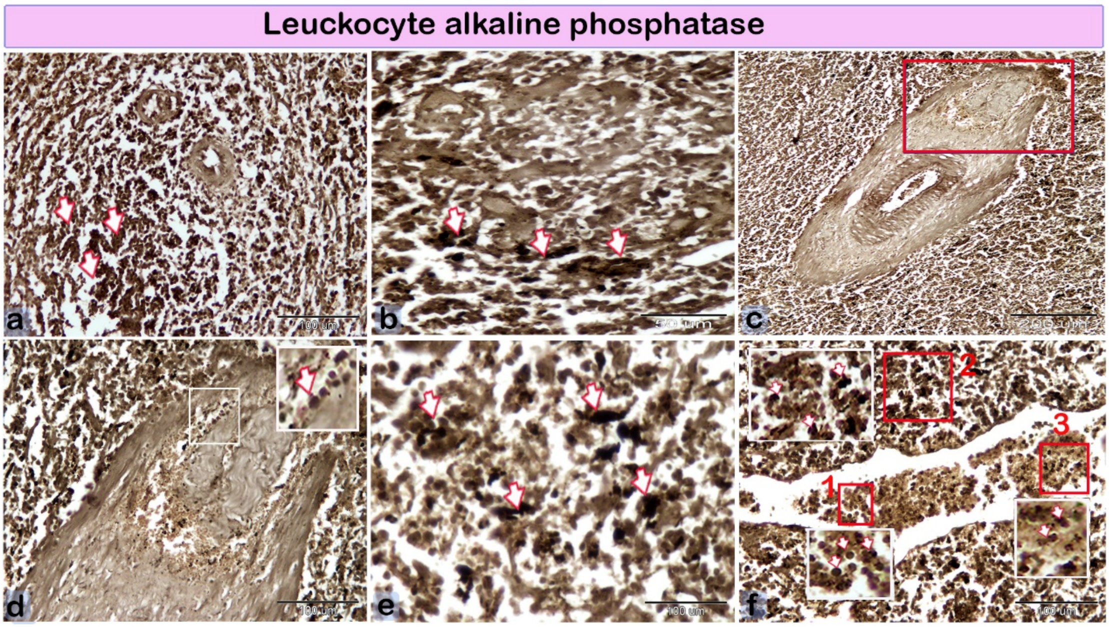

Figure S17. Paraffin sections stained by the Gomori calcium method for alkaline phosphatase activity showing: a: lymphocytes stained positively (arrows). b: Destructed neutrophil and macrophage stained positively (arrows). c and d: Low and higher magnification of splenic trabecula were thickened due to swelled and distended smooth muscular trabecula. Blood vessel inside the splenic trabecula was dilated and engorged with blood. Note: diffuse lymphocytes (arrowheads) and neutrophils (arrows) stained positively with alkaline phosphatase. e: strong positive staining of alkaline phosphatase in the macrophage (arrows) within the red pulp. f: The reaction of alkaline phosphatase in macrophage within the interstitial space (square 2). Note, the reaction in lymphocytes (square 1) and neutrophil in (square 3) within the blood vessels.

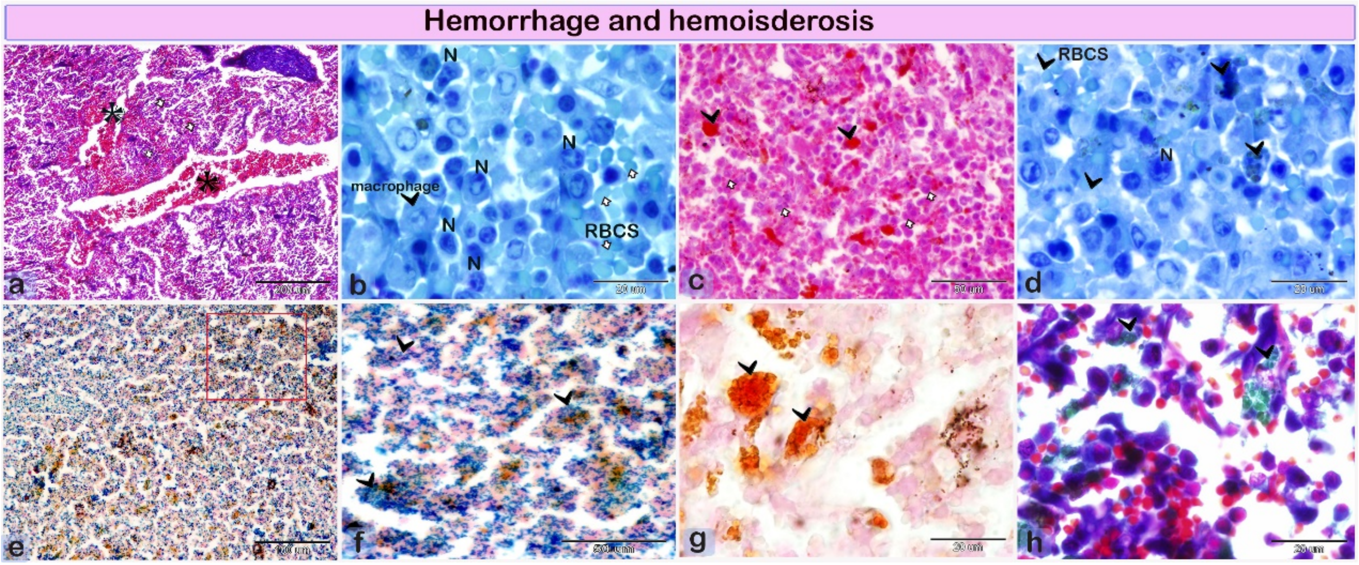

Figure S18. a, c , e–h paraffin sections and b and d, semi-thin sections of spleen showing: a, marked diffuse hemorrhage, the blood vessels are severely dilated and engorged with blood, b-d extravasated hemorrhage in the interstitial tissue (*). Note, N is neutrophil, (macrophage, arrowheads), (RBCs, arrows) and orange lipofuscin pigment (arrowheads), arrows pointed to leukocyte infiltration. In d showing the macrophage with lipofuscin pigment. e: Low and f: high magnification of selected red square showing the diffuse hemosiderosis spots (arrowheads) of dispersed bluish precipitations of iron pigments in the white and red pulp of the spleen. g: Golden brown Lipofuscin pigment (black arrows) stained positively with Long zheil Nielsen's, green staining of granules within the

macrophage. h: Macrophage with green stained granules. a and c: stained by HX and eosin, b and d: stained by toluidine blue, e and f: stained by Prussian blue stain for iron. g: stained with Long Zheil Nielsen; h: stained with Crossmon 's trichrome stain.
